# Aberrant over-expression of COX-1 intersects multiple pro-tumorigenic pathways in high-grade serous ovarian cancer

**DOI:** 10.18632/oncotarget.3860

**Published:** 2015-05-04

**Authors:** Andrew J. Wilson, Oluwole Fadare, Alicia Beeghly-Fadiel, Deok-Soo Son, Qi Liu, Shilin Zhao, Jeanette Saskowski, Md. Jashim Uddin, Cristina Daniel, Brenda Crews, Brian D. Lehmann, Jennifer A. Pietenpol, Marta A. Crispens, Lawrence J. Marnett, Dineo Khabele

**Affiliations:** ^1^ Department of Obstetrics & Gynecology, Vanderbilt University Medical Center, Nashville, TN; ^2^ Department of Pathology, University of California San Diego School of Medicine, La Jolla, CA; ^3^ Department of Medicine, Division of Epidemiology, Vanderbilt University Medical Center, Nashville, TN; ^4^ Vanderbilt-Ingram Cancer Center, Vanderbilt University Medical Center, Nashville, TN; ^5^ Department of Biochemistry & Cancer Biology, Meharry Medical College, Nashville, TN; ^6^ Department of Biomedical Informatics, Vanderbilt University Medical Center, Nashville, TN; ^7^ Vanderbilt Center for Quantitative Sciences, Vanderbilt University School of Medicine, Nashville, TN; ^8^ Department of Biochemistry, Vanderbilt University Medical Center, Nashville, TN; ^9^ Vanderbilt Institute of Chemical Biology, Vanderbilt University Medical Center, Nashville, TN

**Keywords:** high-grade serous ovarian cancer, cyclooxygenase-1, cell migration/invasion, pro-tumorigenic pathways

## Abstract

Cyclooxygenase-1 (COX-1) is implicated in ovarian cancer. However, patterns of COX expression and function have been unclear and controversial. In this report, patterns of COX-1 and COX-2 gene expression were obtained from RNA-seq data through The Cancer Genome Atlas. Our analysis revealed markedly higher COX-1 mRNA expression than COX-2 in high-grade serous ovarian cancers (HGSOC) and higher COX-1 expression in HGSOC tumors than 10 other tumor types. High expression of COX-1 in HGSOC tumors was confirmed in an independent tissue microarray. In contrast, lower or similar expression of COX-1 compared to COX-2 was observed in endometrioid, mucinous and clear cell tumors. Stable COX-1 knockdown in HGSOC-representative OVCAR-3 ovarian cancer cells reduced gene expression in multiple pro-tumorigenic pathways. Functional cell viability, clonogenicity, and migration/invasion assays were consistent with transcriptomic changes. These effects were reversed by stable over-expression of COX-1 in SKOV-3 cells. Our results demonstrate a distinct pattern of COX-1 over-expression in HGSOC tumors and strong association of COX-1 with multiple pro-tumorigenic pathways in ovarian cancer cells. These findings provide additional insight into the role of COX-1 in human ovarian cancer and support further development of methods to selectively target COX-1 in the management of HGSOC tumors.

## INTRODUCTION

Ovarian cancer is the most lethal gynecologic malignancy with high-grade serous ovarian cancer (HGSOC) representing the most common and biologically aggressive histological subtype [1, 2]. Frequent late stage presentation of disease and the high incidence of relapse following standard platinum-based therapy points to an urgent need for more effective diagnostic, preventive, and therapeutic strategies. HGSOC harbor alterations in the tumor suppressor gene, *TP53 (>95%)* and defects in homologous recombination DNA repair genes *(~50%)*, but otherwise are molecularly heterogeneous tumors with no other clear molecular targets.

The cyclooxygenase-1 (COX-1) enzyme was suggested as a potential molecular target in ovarian cancer when it was first reported as a tumor-associated antigen over 20 years ago [3, 4]. COX-1 and COX-2 are rate-limiting enzymes in the early steps of prostaglandin (PG) biosynthesis and convert the fatty acid arachidonic acid (AA) to biologically active PGs and thromboxane A_2_ [5, 6]. Pro-tumorigenic functions of COX-generated PGs include increased tumor cell growth, avoidance of apoptosis, angiogenesis, epithelial-mesenchymal transition (EMT), and promotion of an endothelial immune barrier preventing cytotoxic T cell infiltration into tumors [7–9]. Older paradigms viewed COX-1 as a constitutive ubiquitous “housekeeping” enzyme associated with physiologic PGs, and COX-2 as an inducible enzyme whose over-expression is linked to production of pathophysiological PGs and cancer. In a paradigm shift, our group and others have revealed that COX-1, rarely COX-2, is over-expressed in multiple human and mouse models of ovarian cancer [10–13]. Further, a potential pro-tumorigenic role for COX-1 in ovarian cancer is inferred by the ability of COX-1 inhibitors to suppress ovarian tumorigenesis in these models. In contrast, selective COX-2 inhibitors are relatively ineffective [10–12, 14–17]. Other groups have shown a role for COX-2 and use of COX-2 inhibitors in ovarian tumors and cell lines [18–22]. However, many of the COX-2 studies did not assess COX-1, due to the assumption that it was a “housekeeping” gene. Moreover, most of the previous studies were limited by reliance solely on protein expression of COX enzymes, with potential discrepancies from antibody cross-reactivity. Dependence on COX-1 inhibitors with poor bioavailability and potency to infer tumor cell autonomous effects of COX-1 has also been a limitation. These inconsistent results also suggest temporal, contextual, and tissue-dependent functions, for COX-1 and COX-2, including differences in histological subtype [7].

Conflicting epidemiological studies of COX inhibitors have added to the controversy. A pooled analysis of 12 epidemiological studies revealed that regular use of the COX inhibitors aspirin and non-steroidal anti-inflammatory drugs (NSAIDs) was associated with decreased ovarian cancer risk [23]. Other studies have produced contradictory results [24, 25]. Furthermore, a recent report showed no evidence that aspirin and NSAIDs improve survival in women with ovarian cancer [26]. This survival study was limited by only assessing pre-diagnosis, not post-diagnosis use. All of these studies are limited by the fact that no commercially available potent and selective COX-1 inhibitors exist in the United States. Despite an apparent paradigm shift showing preclinical promise for COX-1 as a molecular target, the precise role of COX-1 in ovarian cancer remains unclear. This lack of clarity and controversial results have hampered the clinical development of COX-1 as a diagnostic, chemopreventive, and therapeutic target. If COX-1 is indeed a viable molecular target in ovarian cancer, these controversies will need to be addressed.

In this study, we used The Cancer Genome Atlas (TCGA) dataset and our own tumor bank to demonstrate that COX-1 gene and protein expression levels are distinctly elevated in HGSOC tumors compared to other tumor types, including other histological subtypes of ovarian cancer. Second, we generated unique, stable isogenic cell line models, shRNA-mediated down-regulation (OVCAR-3) and vector-mediated over-expression (SKOV-3), to demonstrate that down-regulating COX-1 gene expression inhibits multiple pro-tumorigenic pathways in a coordinated fashion. Finally, we validated that knockdown of COX-1 inhibits pro-tumorigenic functions such as cell viability, clonogenicity, and migration/invasion in COX-1 expressing ovarian cancer cells. Taken together, these results address some of the existing gaps in knowledge, establish a conceptual framework for the role of COX-1 in HGSOC tumors, and provide additional support for COX-1 as an attractive molecular target in ovarian cancer.

## RESULTS

### COX-1 has a distinct pattern of over-expression in HGSOC tumors

**Figure 1:**
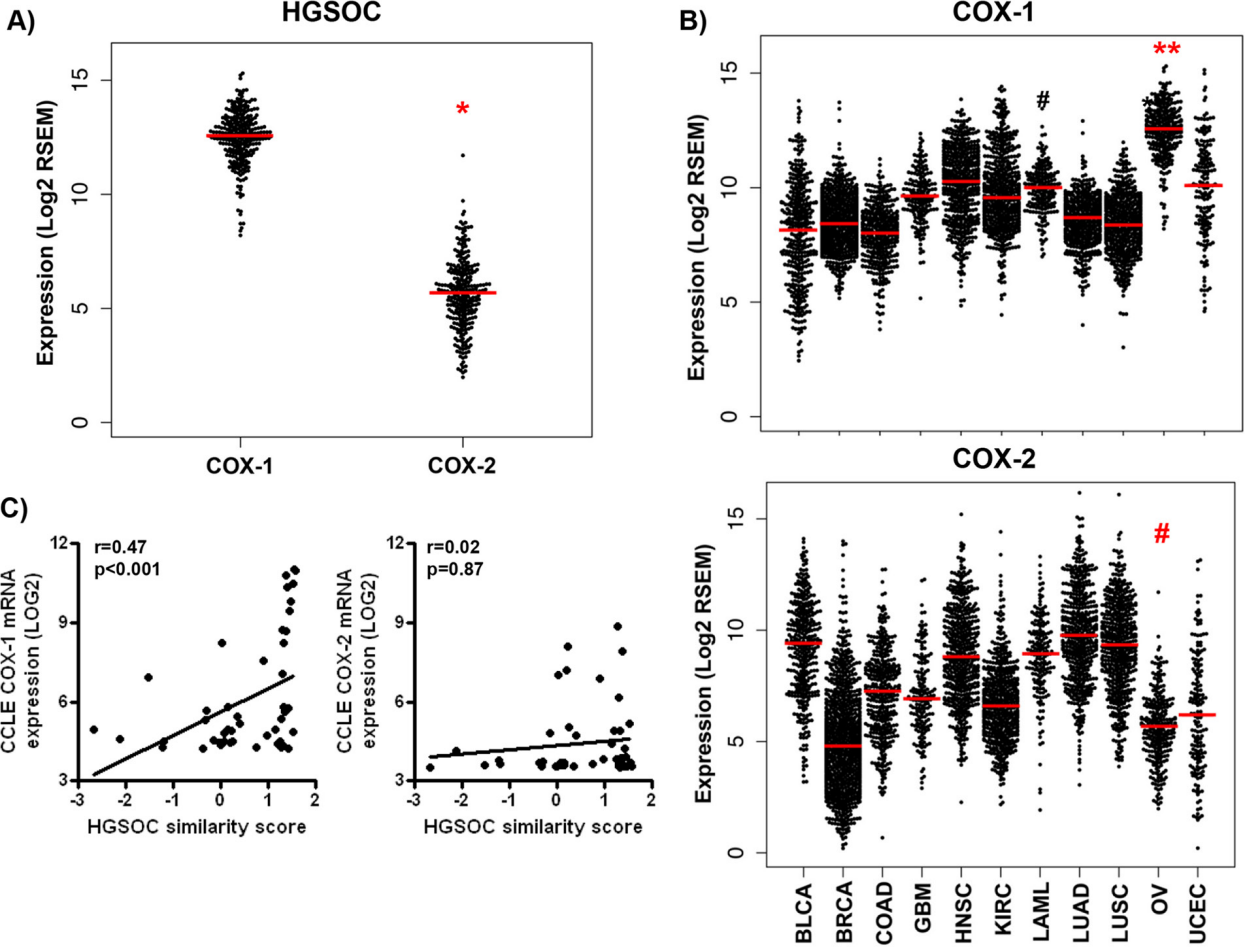
COX-1 mRNA is over-expressed in high-grade serous ovarian tumors and representative cell lines RNA-seq analysis of COX-1 and COX-2 expression in TCGA tumors. RSEM-normalized RNA-seq values for COX-1 and COX-2 for **A.** ovarian serous cancers (OV) and **B.** the full panel of PANCAN tumors were downloaded from cBioPortal. RSEM values were log2-transformed and plotted with the R package beeswarm (median indicated by red bar). *P* values were determined by the Mann-Whitney test; *p < 0.0001 compared to COX-1; **p < 0.0001 compared to all other tumor types; ^#^*p* < 0.01 compared to all other tumor types. Expression of **C.** COX-1 and **D.** COX-2 mRNA in 47 ovarian cancer cell lines extracted from the Broad Institute CCLE repository (http://www.broadinstitute.org/ccle). Raw mRNA data were log2-transformed. Expression was correlated by Spearman correlation with the published HGSOC similarity score for ovarian cancer cells.

First, to determine patterns of expression of *COX-1* and *COX-2* measured by RNA-seq, we extracted data from the TCGA database in which HGSOC tumors are the only histological type of ovarian cancer [27]. COX-1 mRNA levels were significantly higher than those of COX-2 in HGSOC tumors (log2 transformed counts of 12.5 ± 1.2 and 5.6 ± 1.6 respectively, mean ± SD, *p* < 0.0001, Mann-Whitney test) (Figure 1A). Furthermore, COX-1 mRNA was more highly expressed in HGSOC tumors than in any other PANCAN tumor (*p* < 0.0001, Mann-Whitney test) (Figure 1B). In contrast, COX-2 mRNA expression in HGSOC tumors was significantly reduced compared to all other tumor types (*p* < 0.01, Mann-Whitney test) with the exception of BRCA tumors, where lower COX-2 mRNA levels were detected (*p* < 0.0001, Mann-Whitney test).

Next, we examined levels of COX-1 and COX-2 mRNA in ovarian cancer cell lines represented in publically available resources. Microarray data from the NCI 60 cell line repository indicates that the ovarian cancer cell lines, OVCAR-3 and OVCAR-4, which are molecularly similar to HGSOC tumors [28], have the highest basal expression of COX-1 mRNA among all 60 cell lines in the panel (Supplementary Table S1). In contrast, COX-2 mRNA expression is relatively lower in ovarian cancer cells compared to other cell lines (Supplementary Table S2). We observed a similar pattern of effect in a larger panel of cell lines, available from the Broad-Novartis CCLE repository (Figure 1C and Supplementary Figure S1). In 47 unique CCLE ovarian cancer cell lines previously annotated through an HGSOC “similarity score” [29], there was a significant positive association between COX-1 mRNA expression and the HGSOC score, indicative of higher COX-1 expression in cell lines most representative of the serous subtype (Figure 1C). In contrast, there was no significant association between COX-2 and the HGSOC score.

**Figure 2:**
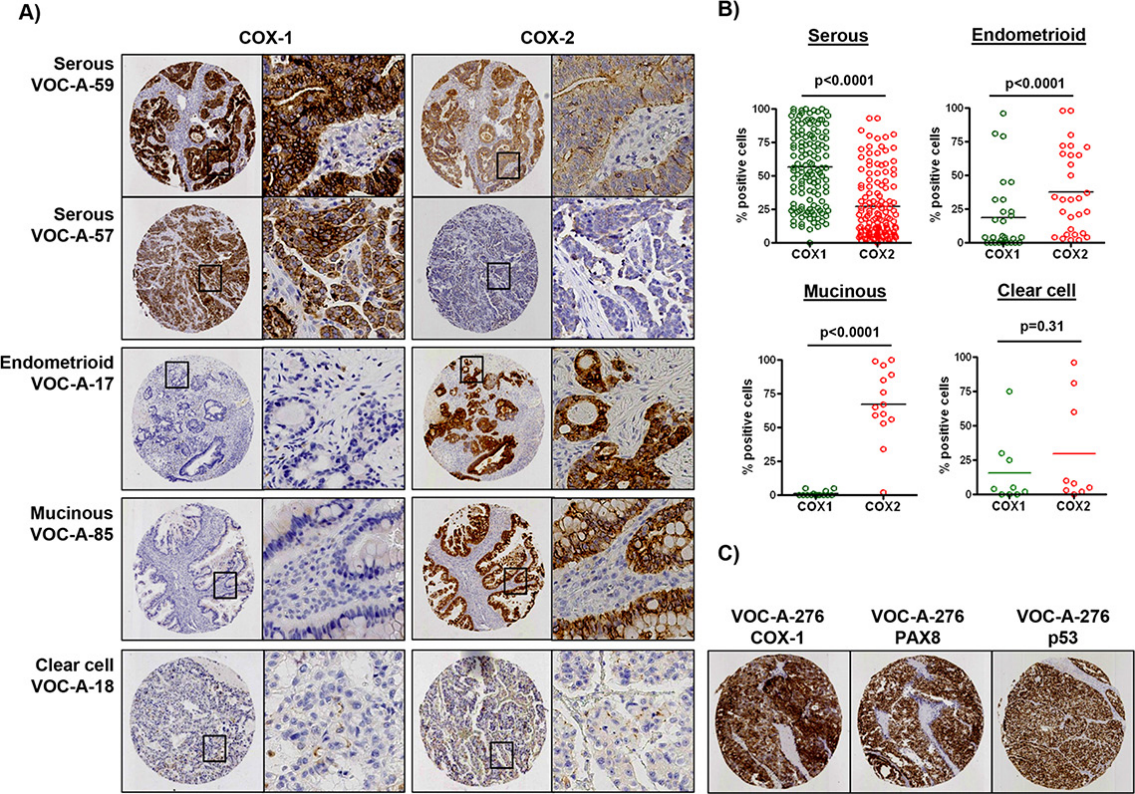
Differential protein expression of COX-1 and COX-2 in ovarian cancer histological subtypes **A.** Representative sections from a TMA of 209 ovarian tumors after immunostaining with COX-1 and COX-2. The percentage of tumor cells positive for COX-1 or COX-2 was determined by automated image analysis. **B.** Plots show the percentage of COX-1 and COX-2 positive tumor cells in serous, endometrioid, mucinous and clear cell tumors. **C.** Co-expression of COX-1, PAX8 and p53 in a representative serous tumor. *P* values were determined by the Mann-Whitney test.

To determine if patterns of COX-1 and COX-2 mRNA expression are similar at the protein level, we performed immunohistochemistry (IHC) staining of a tissue microarray (TMA) of ovarian cancer samples from an independent dataset generated in our laboratory [30] (Supplementary Table S3). Stratified staining data (high, moderate, or weak) for COX-1 and COX-2 in low-grade (grade 1) and high-grade (grade 2/3) serous, endometrioid, mucinous and clear cell tumors, and corresponding statistical analysis, are shown in Table 1. We found that COX-1 protein was moderately to highly expressed in 99% of high-grade tumors, where it was confined to the epithelium, and was co-expressed with the HGSOC markers PAX8 and mutant p53 (Figure 2A–2C). In contrast, COX-2 was located in both the epithelium and stroma, had wide variation in expression levels in high-grade tumors, and was highly expressed in endometrioid and mucinous tumors (Figure 2A–2B). COX-1 expression was significantly higher than COX-2 in high-grade tumors and across all serous tumors compared to endometrioid, mucinous and clear cell tumors. In the relatively small number of representative clear cell tumors, expression of COX-1 and COX-2 was not significantly different.

**Table 1:** Clinical characteristics and expression of COX-1 and COX-2 in ovarian cancer tumors

Ovarian Carcinomas	COX-1 Staining		COX-2 Staining		*P*-value*
	*N*	%	*N*	%	
By Histologic Subtype					
**Serous**^a^					<**0.001**
High	77	58.8	27	20.6	
Moderate	53	40.5	61	46.6	
Weak	1	0.8	43	32.8	
**Endometrioid**^b^					**0.021**
High & Moderate	10	40.0	10	76.0	
Weak	15	60.0	15	24.0	
**Mucinous**^c^					<**0.001**
High & Moderate	0	0.0	13	92.9	
Weak	14	100.0	1	7.1	
**Clear cell**					0.620
High & Moderate	2	22.2	4	44.4	
Weak	7	77.8	5	55.6	
**Serous Tumors, By Grade**					
**High Grade Serous (Grade 2&3)**					<**0.001**
High & Moderate	111	99.1	71	63.4	
Weak	1	0.9	41	36.6	
**Low Grade Serous (Grade 1)**					0.490
High & Moderate	19	100.0	17	89.5	
Weak	0	0.0	2	10.5	

* *P*-value from the χ^2^ test, except when expected cell counts were ≤5, in which case Fishers’ Exact test was used. High = >50% positive tumor cells; Moderate = 10–50% positive tumor cells; Weak = < 10% positive tumor cells. Missing data:

a 9/140 serous tumor spots missing from TMA;

b 2/27 endometrioid tumor spots missing;

c 1/15 mucinous tumor spots missing.

In a subset of HGSOC tumors from which quality RNA was extracted from paraffin blocks, we confirmed robust COX-1 mRNA expression (Supplementary Figure S2A). Further, there was a strong association between mRNA and protein expression of COX-1 (Spearman r = 0.7, *p* < 0.0001) (Supplementary Figure S2B). A similar association was observed for COX-2 mRNA and protein levels (Spearman r = 0.69, *p* < 0.0001).

We evaluated patterns of COX-1 protein expression by Western blot in well-established ovarian cell lines. We confirmed that cell lines representative of HGSOC tumors such as OVCAR-3 and OVCAR-4 had elevated COX-1 protein levels compared to SKOV-3 cells, which are poorly representative of HGSOC tumors [28, 29] (Supplementary Figure S2C). COX-1 was also highly expressed in cell lines from spontaneously derived (ID-8) [31] and genetically engineered mouse models ovarian cancer (Supplementary Figure 2C) [32, 33]. In sharp contrast, protein expression of COX-2 was low to undetectable in all cell lines examined (Supplementary Figure S2C).

### Knockdown of COX-1 gene expression inhibits multiple pro-tumorigenic pathways *in vitro*

**Figure 3:**
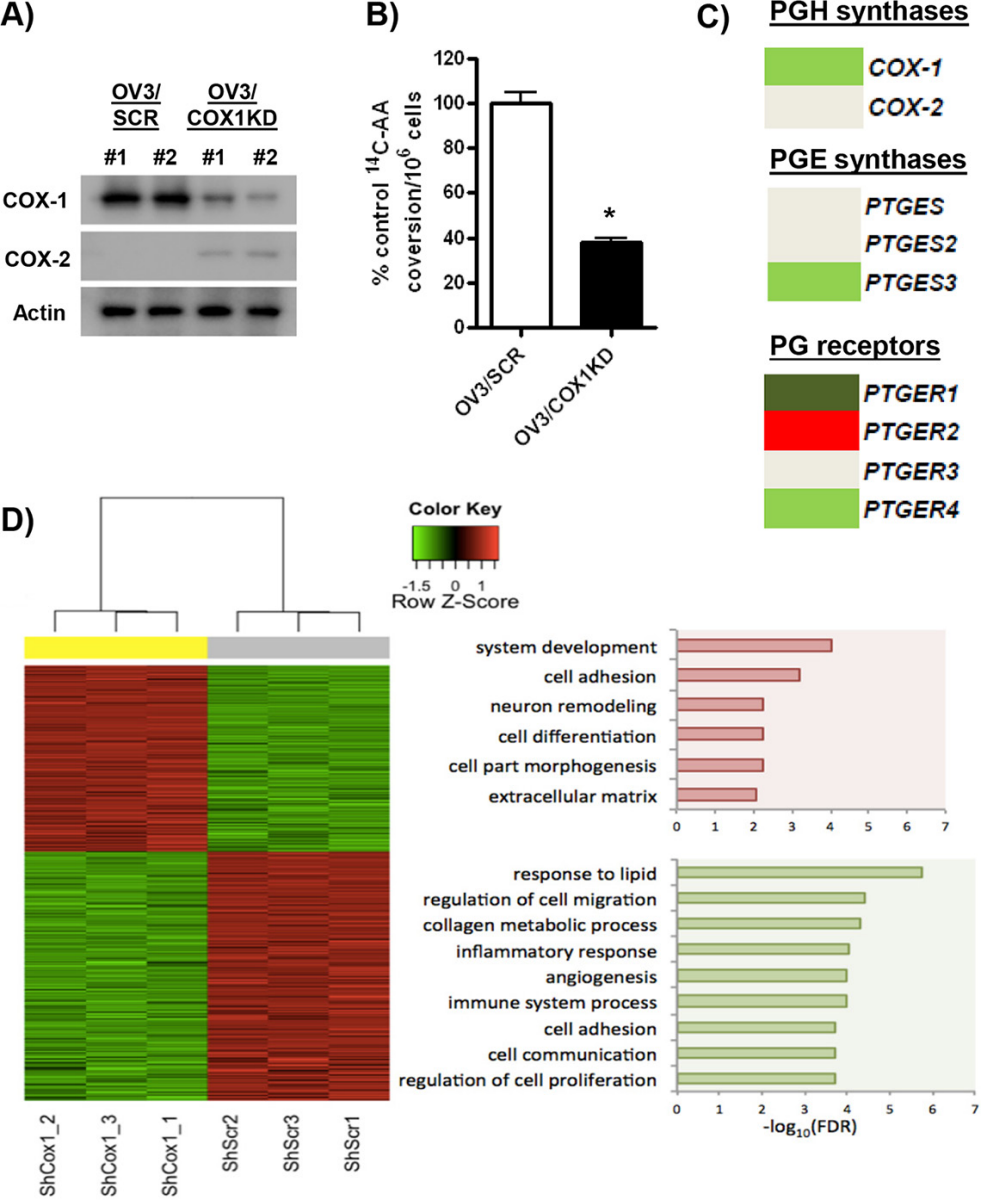
Analysis of genes involved in PG regulation and cellular pathways regulated by COX-1 knockdown **A.** Protein levels of COX-1, COX-2 and actin in OVCAR-3 clones stably transfected with shRNA targeting COX-1 (OV3/COX1KD) or control scrambled ShRNA (OV3/SCR). **B.** Conversion of ^14^C-arachidonic acid (^14^C-AA) to prostaglandin products in OV3/SCR and OV3/COX1KD cells after 30 min stimulation in serum-free medium. Results are expressed as a percentage of conversion in control OV3/SCR cells per 10^6^ cells. **C.** RNA-seq analysis of expression of PG receptors and PGE synthases in OV3/COX1KD cells compared to control OV3/SCR cells. Light green (log2 fold change < −0.5, FDR < 0.01), dark green (log2 fold change < −1.5, FDR < 0.01), light red (log2 fold change>0.5, FDR < 0.01), dark red (log2 fold change>1.5, FDR < 0.01), DeSeq2 analysis. **D.** WebGestalt functional enrichment analysis of RNA-seq data.

We have shown previously that COX-1 inhibitors suppress ovarian cancer cell growth and that short-term down-regulation of COX-1 gene expression activates p21 and inhibits cell proliferation [14, 34]. To investigate the effects of COX-1 inhibition further, we used stable isogenic cell lines to interrogate the effects of genetic disruption of COX-1 in ovarian cancer. First, in COX-1 positive OVCAR-3 cells, we created cell lines stably expressing short hairpin RNA (shRNA) targeting COX-1 (OV3/COX1KD). Robust down-regulation of COX-1 expression in two clones (OV3/COX1KD #1, #2) compared to control cells stably transfected with scrambled shRNA (OV3/SCR) was confirmed at the protein level (Figure 3A). OV3/COX1KD clone, #2 was selected as these cells displayed the most efficient down-regulation of COX-1 protein expression. We then confirmed that OV3/COX1KD cells were markedly less responsive to AA stimulation in ^14^C-AA metabolism studies measuring PG production (Figure 3B).

To investigate the underlying molecular consequences of COX-1 down-regulation, we measured global gene expression by RNA-seq in the COX-1 proficient OVCAR-3/SCR cells compared to the most efficient COX-1 knockdown derived from OV3/COX1KD clone #2. First, we confirmed significant down-regulation of COX-1 mRNA levels in OV3/COX1KD cells (Figure 3C and Supplementary Figure S3A–S3B). A slight increase in COX-2 protein expression was detected in our OV3/COX1KD clones (Figure 3A), although this effect was not significant at the mRNA level (Figure 3C and Supplementary Figure S3A&S3B). Cellular PGE levels are regulated by COX proteins, which convert AA to PGH, and by PGE synthases (PTGES), which convert PGH to PGE [5, 6]. RNA-seq analysis demonstrated that cytosolic PTGES3 was significantly down-regulated in OV3/COX1KD cells (Figure 3D), suggesting both catalytic steps in the PG biosynthetic pathway were reduced following COX-1 knockdown, accounting for the observed reduction in PG levels following AA stimulation (Figure 3B). In contrast, the microsomal (PTGES) and membrane-bound (PTGES2) forms were not altered (Figure 3D). Since cellular response to PGE_2_ is dependent on PG receptors (PTGER1-4), we analyzed their expression in our isogenic cells. As shown in Figure 3C, two isoforms (PTGER1 and PTGER4) showed significant down-regulation, PTGER2 was up-regulated, and PTGER3 did not change.

WebGestalt functional enrichment analyses confirmed significant down-regulation of multiple pro-tumorigenic pathways, such as cell proliferation growth, angiogenesis, cell migration/invasion, inflammation and immune regulation in COX-1 knockdown cells (Figure 3D). Analysis of individual gene members of these, and intersecting pro-tumorigenic, pathways revealed coordinated regulation promoting an anti-tumor phenotype.

**Figure 4:**
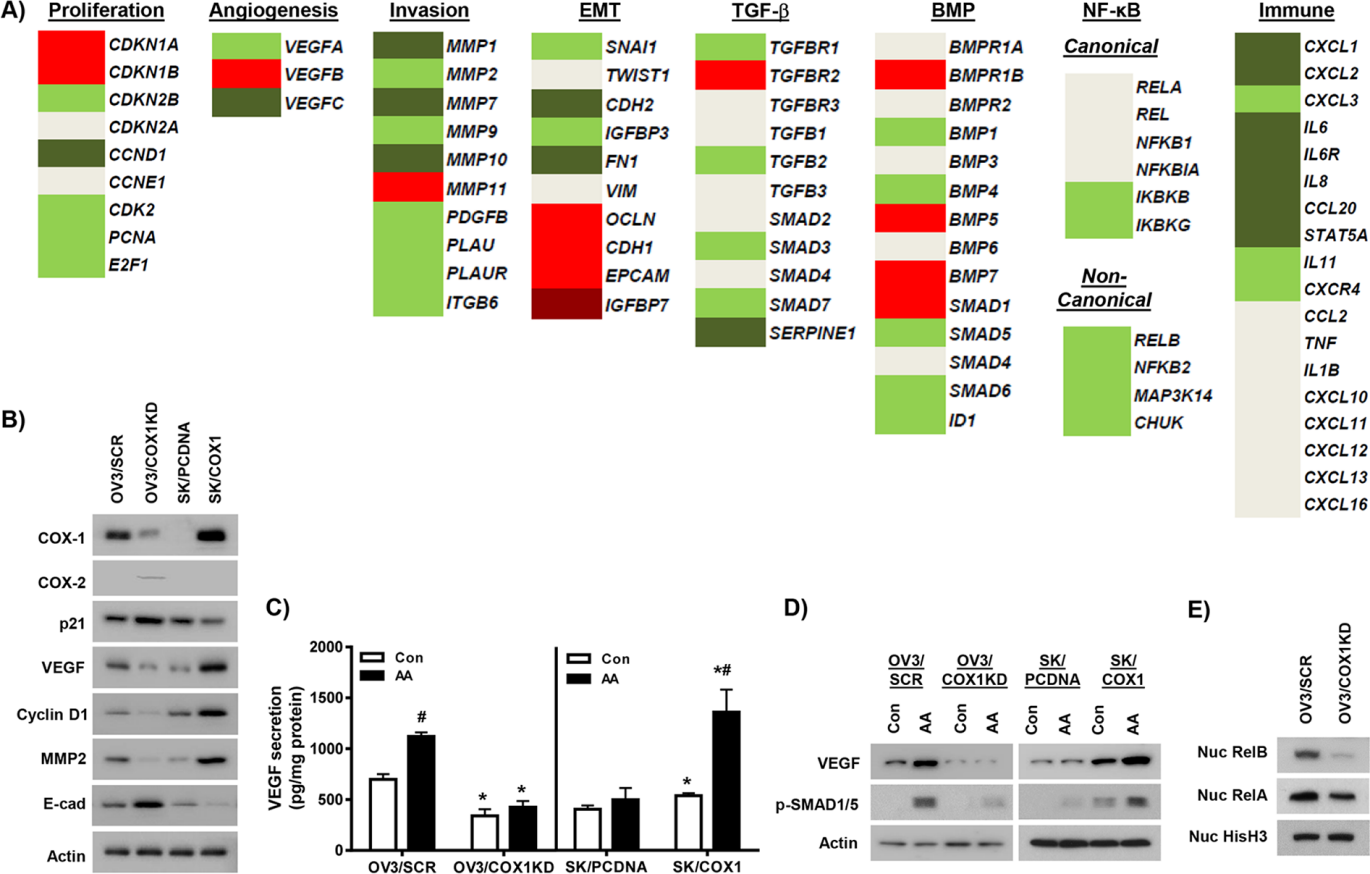
Individual genes regulated by COX-1 knockdown **A.** RNA-seq analysis of individual gene members of cellular processes, proliferation, angiogenesis, EMT, cell invasion, TGF-β and BMP signaling, NF-κB signaling and inflammation/immune processes in OV3/COX1KD cells compared to control OV3/SCR cells. Light green (log2 fold change < −0.5, FDR < 0.01), dark green (log2 fold change < −1.5, FDR < 0.01), light red (log2 fold change>0.5, FDR < 0.01), dark red (log2 fold change>1.5, FDR < 0.01), DeSeq2 analysis. **B.** Western blot analysis of protein expression of selected altered genes in expression in OV3/COX1KD cells compared to OV3/SCR cells. Effects of COX-1 over-expression on protein expression of these genes in SK/COX1 cells compared to SK/PCDNA cells were also determined. Actin was used as loading control. **C.** Secretion of VEGF into serum-free medium after 24 h incubation with and without exposure to 20 μM arachidonic acid (AA). Values are mean + SEM of 3 independent experiments. *p < 0.01 relative to corresponding control cell line; ^#^*p* < 0.01 compared to control treatment in AA stimulation experiments, Student's *t* test. **D.** Western blot analysis of cellular VEGF and phospho-Smad1/5 expression following 24 h stimulation with AA (20 μM). **E.** Expression of RelB and RelA in nuclear extracts from OV3/COX1KD and OV3/SCR cells. Nuclear histone H3 levels were used as loading control.

*Cell proliferation* COX-1 knockdown was associated with down-regulation of multiple genes promoting cell cycle progression and/or DNA synthesis, including *CCND1, CDK2, PCNA*, and *E2F1*, and up-regulation of the cell cycle inhibitors, *CDKN1A* (p21) and *CKN1B* (p27) (Figure 4A–4B).

*Angiogenesis and cell migration/invasion* Individual genes involved in promoting angiogenesis (such as *VEGFA* and *VEGFC*) (Figure 4A), and cell invasion/migration (such as *MMP1, MMP2, MMP7, MMP9, PLAU, PLAUR* and the *ITGB6* integrin) [35–37] were significantly down-regulated in the COX-1 knockdown cells (Figure 4A–4B), supportive of a role for COX-1 in promoting tumor metastasis. We confirmed our data for VEGF by demonstrating that basal levels of VEGF secretion (Figure 4C) were lower in COX-1 deficient OVCAR-3 cells, which also showed attenuated VEGF secretion and cellular expression in response to stimulation with AA (Figure 4C–4D).

*Other pro-tumorigenic processes/signaling pathways* Epithelial-mesenchymal transition (EMT) is another key characteristic of tumor metastasis [38]. Established regulators of EMT, such as *SNAI1* (SNAIL), *FN1* (fibronectin), *CDH2* (N-cadherin) and *IGFBP3* (insulin-like growth factor binding protein 3) [38, 39], were down-regulated by COX-1 knockdown. In contrast, the negative regulators of EMT promoting cell-cell adhesion, such as *CDH1* (E-cadherin), *EPCAM* and *OCLN* (occludin) [38], and the *IGFBP7* antagonist of IGFBPs [40], were significantly up-regulated in OV3/COX1KD cells (Figure 4A&4B).

We next examined key genes involved in the transforming growth factor-β (TGF-β) and bone morphogenetic protein (BMP) signaling pathways, which are known to promote EMT among other pro-tumorigenic actions [38, 41]. In COX-1 knockdown cells, there were variable effects on expression of receptors for TGF-β (*TGFBR1, TGFBR2, TGFBR3*) and BMP (*BMPR1A, BMPR1B, BMPR2*) (Figure 4A). In cancer cells, the TGFB2 and BMP2&4 ligands are implicated with promoting EMT, whereas BMP5 and BMP7 are inhibit basal and/or TGF-β-induced EMT [41]. Interestingly, *BMP4* was down-regulated, and *BMP5* and *BMP7* were both up-regulated in OV3/COX1KD cells, consistent with the anti-EMT phenotype observed. To determine overall effects on pathway activity, we first examined expression of well-established targets: *SMAD7* and *SERPINE1*, and *SMAD6* and *ID1*, are established targets of TGF-β and BMP signaling, respectively [42, 43]. As shown in Figure 4A, both *SMAD7/SERPINE1*, and *SMAD6/ID1* were down-regulated in OV3/KD1 cells. We also confirmed that expression of multiple other established BMP and TGF-β targets were down-regulated in our RNA-seq database [44–46]. (Supplementary Figure S4A&S4B). Second, we assessed BMP pathway function through Western blot analysis of phosphorylated SMAD1/5 (p-SMAD1/5). SMADs1&5 are phosphorylated by BMP receptor activation and translocate to the nucleus in a complex with SMAD4 to regulate gene transcription [42]. Basal levels of p-SMAD1/5 were not detectable in unstimulated OV3/SCR or OV3/COX1KD cells. However, stimulation with AA markedly induced p-SMAD1/5 expression in OV3/SCR cells, but not OV3/COX1KD cells (Figure 4D). These results implicate COX-1 and PGs in this pro-tumorigenic pathway.

Finally, we examined expression of key components of the NF-κB signaling pathway, which regulates multiple pro-tumorigenic processes [47, 48]. Several individual genes involved in NF-κB signaling were down-regulated following COX-1 knockdown (Figure 4A). Strikingly, we showed coordinated down-regulation of expression of key mediators of the non-canonical pathway, *RELB, NFKB2* (p100/p52), *CHUK* (IKKα) and *MAP3K14* (NF-κB-inducing kinase) [47]. We then assessed the nuclear expression of RelB and RelA/p65, which is implicated in mediating gene transcriptional effects of canonical and non-canonical NF-κB signaling, respectively. As shown in Figure 4E, there were reduced levels of nuclear RelB, and to a lesser extent RelA/p65, in COX-1 knockdown cells, consistent with inhibition of NF-κB signaling. Furthermore, expression of multiple established targets of NF-κB, identified using the Boston University Biology resource http://www.bu.edu/nf-kb/gene-resources/target-genes/, were down-regulated in COX-1 knockdown cells (Supplementary Figure S4C).

Consistent with the observed down-regulation of NF-κB signaling, inflammation and immune process pathways were also reduced in COX-1 knockdown cells. Expression levels of multiple genes encoding for cytokines or cytokine receptors over-expressed in tumor cells or ascites fluid, such as *CXCL1, CXCL2, CXCL3, IL8, IL6, IL6R*, and *CXCR4* [49–51] were reduced in OV3/COX1KD cells (Figure 4A).

We demonstrated a similar pattern of expression of a subset of the above-mentioned genes in COX-1 knockdown cells at the protein level (Figure 4B). We also tested the effects of COX-1 knockdown on gene expression in a second HGSOC-like ovarian cancer cell line, OVCAR-4, with relatively high COX-1 expression [28, 29]. As shown in Supplementary Figure S5A, we identified two distinct combinations of siRNA duplexes that robustly inhibited COX-1 expression in these cells. Consistent with the results in OVCAR-3 cells, COX-1 down-regulation induced p21 and E-cadherin, and inhibited VEGF, protein expression in OVCAR-4 cells (Supplementary Figure S5A).

To complement our COX-1 knockdown studies, we stably over-expressed COX-1 or empty vector in COX-1 deficient SKOV-3 cells (SK/COX1 and SK/PCDNA, respectively) [14]. SK/COX1 robustly expressed both COX-1 protein (Figure 4B) and mRNA (Supplementary Figure S3B), and displayed increased radiolabeled prostaglandin production following ^14^C-AA stimulation compared to SK/PCDNA cells (Supplementary Figure S3C). COX-1 over-expression in SK/COX1 cells reversed mRNA and protein expression of pro- and anti-tumorigenic genes compared to COX-1 knockdown in OVCAR-3 and OVCAR-4 cells (Figure 4B and Supplementary Figure S3B). SK/COX1 cells have higher basal VEGF levels than SK/PCDNA cells (Figure 4C), but still displayed increased secretion and cellular levels of VEGF following AA exposure (Figure 4C–4D). SK/COX1 cells also showed higher basal levels of p-SMAD1/5 than SK/PCNA cells, and displayed increased responsiveness to AA stimulation (Figure 4D). These results suggest high COX-1 expression was associated with elevated BMP pathway activity.

### COX-1 promotes ovarian cancer cell growth and migration/invasion *in vitro*

**Figure 5:**
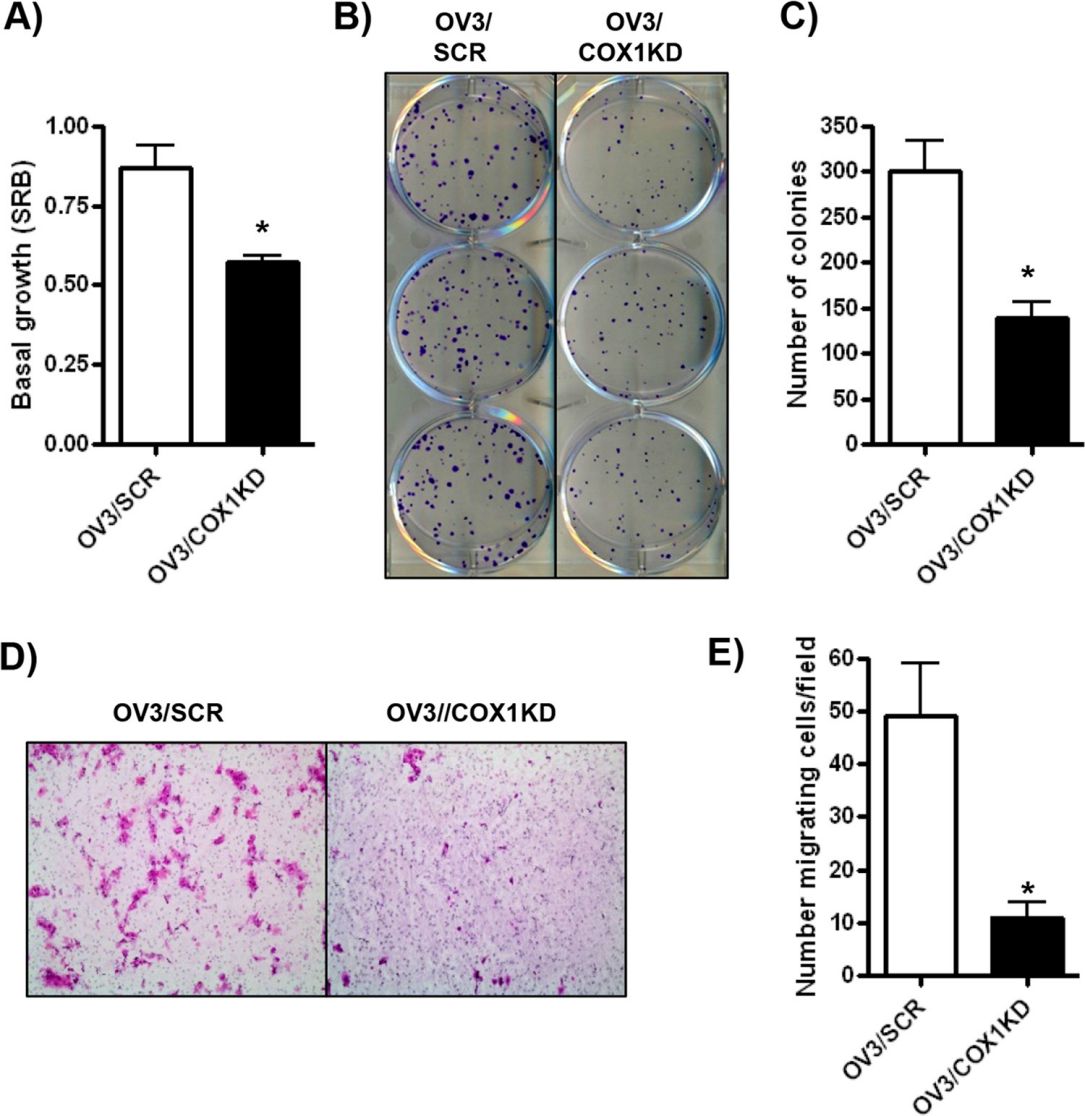
COX-1 knockdown in ovarian cancer cells promotes an anti-tumorigenic cellular phenotype **A.** SRB cell viability assay of OV3/SCR and OV3/COX1KD cells after 72 h growth. **B–C.** Colony forming assay of OV3/SCR and OV3/COX1KD cells. 500 cells were seeded per cell line and colonies allowed to grow for 14 days. **D–E.** Invasion through Matrigel by OV3/SCR and OV3/COX1KD cells after 48 h. Values are mean + SEM of 3 independent experiments. 5 fields were counted per cell line at x20 objective. *p < 0.01 relative to OV3/SCR cells, Student's *t* test.

We used our knockdown and over-expression models to evaluate the cellular consequences of COX-1 expression in ovarian cancer. COX-1 depleted cells (OV3/COX1KD #2) displayed a 50% reduction in cell viability/proliferation at 72 hours compared to scrambled control (OV3/SCR) cells in sulfurhodamine B (SRB) cell viability assays (Figure 5A). The ability of COX-1 knockdown cells to form colonies from single cells was also markedly reduced (Figure 5B–5C). Similar inhibitory effects on cell viability and growth were observed in an additional COX-1 knockdown clone (OV3/COX1KD#1) (Supplementary Figure S6A–S6C), and with transient siRNA-mediated COX-1 in OVCAR-4 cells (Supplementary Figure S5B–S5D). Since we also identified cell invasion/migration as a pathway significantly reduced in COX-1 knockdown cells in our RNA-seq experiments, we determined the role of COX-1 expression in an *in vitro* cell migration/invasion assay. Migration of COX-1 depleted OV3/COX1KD cells through Matrigel-coated transwell was significantly inhibited compared to OV3/SCR cells (Figure 5D–5E).

**Figure 6:**
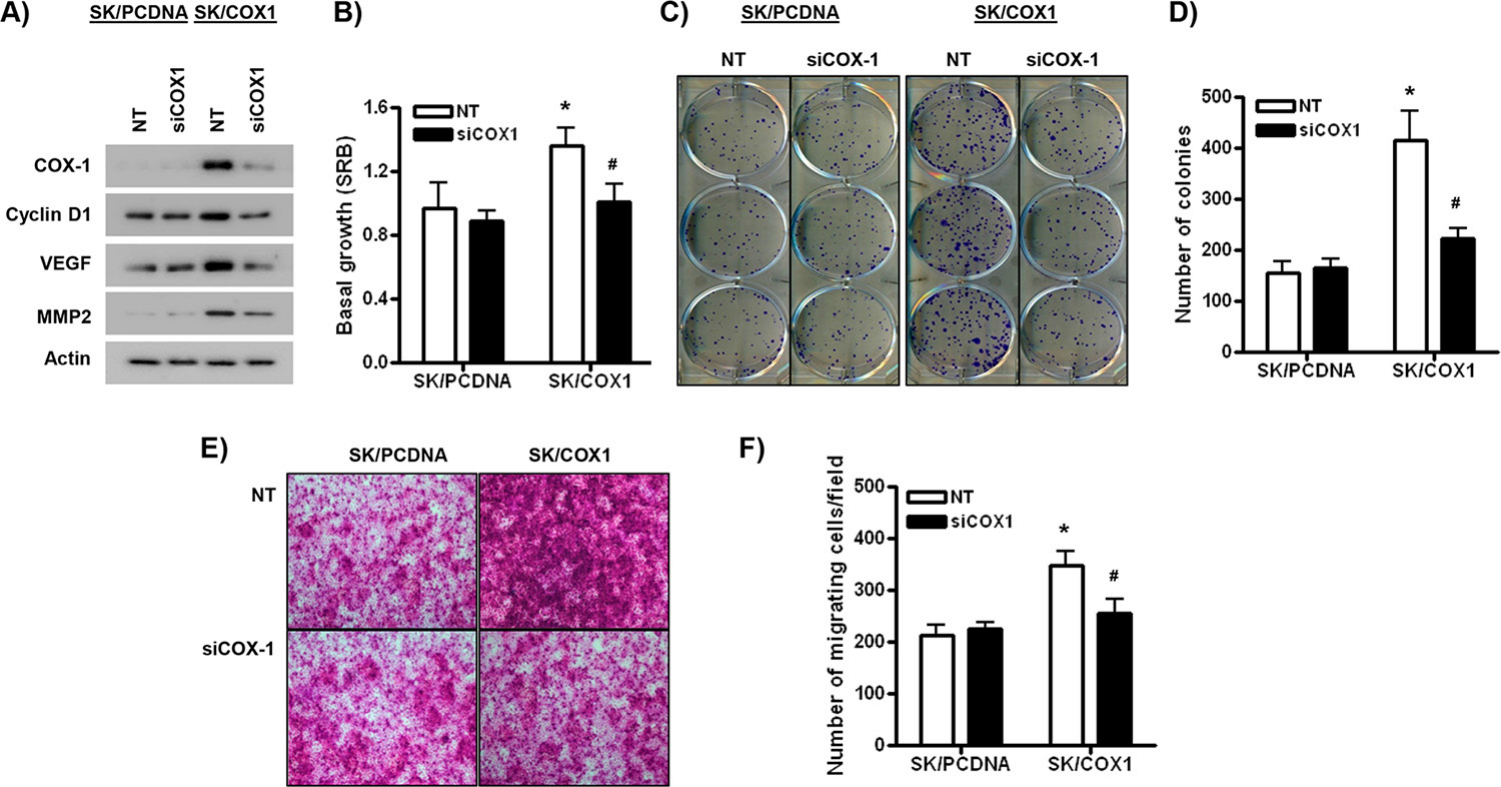
COX-1 over-expression in ovarian cancer cells is pro-tumorigenic **A.** Western blot analysis of SK/PCDNA or SK/COX1 cells transfected with COX-1-targeting siRNA (siCOX1) or control, non-targeting (NT) siRNA (100 nM) for 48 h. **B.** SRB cell viability assay of SK/PCDNA and SK/COX1 cells transfected with siCOX1 or NT (20 nM) after 72 h growth. **C–D.** Colony forming assay of SK/PCDNA and SK/COX1 cells transfected with siCOX1 or NT (20 nM). 400 cells were seeded per cell line treatment and colonies allowed to grow for 7 days growth in low-serum containing medium. **E–F.** Invasion through Matrigel by SK/PCDNA and SK/COX1 cells transfected with siCOX1 or NT (20 nM) after 24 h. 5 fields were counted per cell line/treatment at x20 objective. Values are mean + SEM of 3 independent experiments. *p < 0.01 relative to SK/PCDNA cells, #*p* < 0.01 relative to NT-treated SK/COX1 cells, Student's *t* test.

To validate the specificity of the functional effects of COX-1, we performed experiments where COX-1 over-expression in SK/COX1 cells was reversed by siRNA-mediated knockdown of COX-1. In control, non-targeting (NT) siRNA-transfected cells, SK/COX1 cells displayed higher basal protein levels of COX-1, cyclin D1, VEGF, and MMP2 compared to SK/PCDNA (Figure 6A). The stimulatory effects of COX-1 over-expression on the expression of cyclin D1, VEGF, and MMP2 were efficiently inhibited by siRNA-mediated knockdown of COX-1 in SK/COX1 cells (Figure 6A). Furthermore, NT siRNA-transfected SK/COX1 cells displayed higher levels of basal cell growth and viability, clonogenicity and invasion compared to SK/PCDNA cells treated with NT siRNA (Figure 6B–6F). These results are consistent with the increased expression of molecular markers of proliferation and migration/invasion observed in SK/COX1 cells (Figures 4B–6A and Supplementary Figure S3B). Importantly, the stimulatory effects of COX-1 over-expression on cell growth and viability, clonogenicity, and migration/invasion were dependent on COX-1, as they were markedly attenuated by siRNA-mediated COX-1 knockdown in SK/COX1 cells (Figure 6B–6F).

## DISCUSSION

In this study, we used multiple methods to gain additional insight into the role of COX-1 gene expression and function in human ovarian cancer. First, we uncovered a distinct pattern of COX-1 up-regulation in HGSOC tumors at gene and protein levels of expression through large-scale analyses of patterns of COX-1 and COX-2 expression in ovarian cancer from public databases and our own tumor bank. Second, we interrogated representative ovarian cancer cell lines with stable genetic knockdown of COX-1 to demonstrate that along with down-regulation of PG signaling, multiple pro-tumorigenic pathways, including proliferation, angiogenesis, migration/invasion, inflammation, and immune processes were down-regulated in a coordinated manner. Individual gene expression changes in EMT, TGF-β, BMP, and NF-κB signaling in COX-1 knockdown cells were consistent with inhibition of these pro-tumorigenic pathways. Finally, we performed functional assays to confirm that a reduction in cell viability, clonogenicity, and migration/invasion in the isogenic cell lines were consistent with the transcriptomic changes. Together, these results provide support for a pro-tumorigenic role for COX-1 in ovarian cancer, particularly HGSOC tumors.

The main strength of this study is the large-scale, quantitative analysis of RNA-seq data for COX-1 and COX-2 gene expression that has been absent in previous studies. The striking pattern of COX-1 over-expression in HGSOC tumors distinguishes these tumors from other tumor types represented in the TCGA. The underlying reason for COX-1 mRNA up-regulation in HGSOC tumors is not known. However, epigenetic mechanisms are implied, because *COX-1* is rarely mutated or amplified in HGSOC tumors [27]. Furthermore, we have also shown a clear enrichment of COX-1 expression above that of COX-2 in a majority of HGSOC tumors compared to other histological subtypes of ovarian cancer in a large TMA. Strong correlation between mRNA and protein expression levels of COX-1 in representative HGSOC tumors is another strength of the study. Although the relatively small numbers of low-grade serous, endometrioid, mucinous and clear cell tumors on our TMA is a limitation, the distribution of ovarian tumor types in this study is in line with the distribution of ovarian cancer in the population [1, 2]. Subsequent independent studies with larger numbers of histological subtypes of ovarian cancer are needed to validate our findings.

One potential limitation of the mRNA expression studies is that the tumors evaluated from the TCGA database and our tumor bank were not microdissected. Although the tissue samples contained greater than 80% tumor per sample, contamination from cells in the stroma/tumor microenvironment such as inflammatory cells could have influenced our results. Despite this potential limitation, immunostaining results of intact tissue demonstrated tumor-specific expression of COX-1 and expression of COX-2 in both tumors and stroma, which is consistent with previous observations [18, 52].

Previous reports have shown that COX-1, not COX-2, is up-regulated in multiple genetically engineered mouse models of ovarian cancer [32, 33], suggesting that COX-1 has a pro-tumorigenic role. In this report, we have confirmed that COX-1, rarely COX-2, protein expression is elevated in genetically engineered models of ovarian cancer with a variety of pro-tumorigenic alterations. Interestingly, all share defects in p53,which is the most common molecular alteration in HGSOC tumors [27]. Thus, these models could be used for investigating specific interactions between COX-1 specific pro-tumorigenic pathways in HGSOC.

Our comprehensive evaluation of the stable isogenic COX-1 ovarian cancer cell lines supports a cell autonomous role of COX-1 in promoting a pro-tumorigenic phenotype in ovarian cancer. A likely mechanism by which COX-1 promotes pro-tumorigenic signaling is through PG production [7–9]. In this study, we demonstrated that overall levels of PGs were reduced in COX-1 knockdown cells. This result reflects down-regulation of AA conversion to PGH (via COX-1) and PGH conversion to PGE via PG receptors. Two out of four PG receptor isoforms were also down-regulated (PTGER1&4). PTGER2 and PTGER4 have been most studied in ovarian cancer and implicated in promoting growth and pro-tumorigenic cytokine production [53–55]. However, the overall influence of PG receptor status remains unclear since the PTGER2 isoform was up-regulated in COX-1 knockdown cells. Our analysis also demonstrated that knockdown of COX-1 gene expression inhibits multiple pro-tumorigenic pathways *in vitro*. Of the pro-tumorigenic pathways, NF-κB stands out as a known target of COX enzymes in ovarian cancer [56] and is an important link between many of the pathways that intersect the COX-1/PG axis in ovarian cancer cells [47, 48]. Although these results will need further validation *in vivo*, they provide additional evidence for a pro-tumorigenic role of COX-1 in ovarian cancer.

Our studies using the isogenic COX-1 cell lines are in line with previous studies showing that COX-1 inhibitors are more effective than COX-2 inhibitors in suppressing tumor growth in preclinical ovarian cancer models [10–12, 14–17]. Our group and others have shown that aspirin, a stronger COX-1 than COX-2 inhibitor, and SC-560, a potent COX-1 selective inhibitor [57], suppress tumor growth in COX-1-overexpressing cell culture and mouse models of ovarian cancer [10–12, 14–17]. Even at low doses, aspirin is a potent inhibitor of COX-1 in pre-systemic circulation and an irreversible inhibitor of COX-1 in anucleated platelets [58]. However, aspirin has only short-term reversible inhibition of COX-1 and COX-2 in nucleated cells [59] and SC-560 is not clinically suitable due to poor bioavailability [57]. Thus, the controversies in the epidemiological literature regarding the role of COX inhibitors aspirin and NSAIDs in ovarian cancer could be due to the lack of available potent and selective COX-1 inhibitors in the clinic. Our results provide support for the development of new COX-1 selective compounds.

Novel COX-1 selective compounds are now in preclinical development [60–62]. To test emerging COX-1 compounds for therapeutic and diagnostic purposes, we will pursue future studies using our OVCAR-3 and SKOV-3 isogenic cell lines as xenografts. These studies will be designed to compare differences in basal growth rates of tumors originating from high and low COX1-expressing cells, confirm molecular changes observed in cultured cells, and importantly, validate the specificity of emerging COX-1 inhibitors. These models will be important tools for designing COX-1 tumor-directed diagnostics and therapeutics. We acknowledge that COX-1 is expressed in a broad spectrum of tissues and has important physiologic functions and toxicities will need to be addressed in the development of systemic COX-1 inhibitors. However, as suggested by recent preclinical mouse models, COX-1 selective compounds have been well-tolerated [60–62].

**Figure 7:**
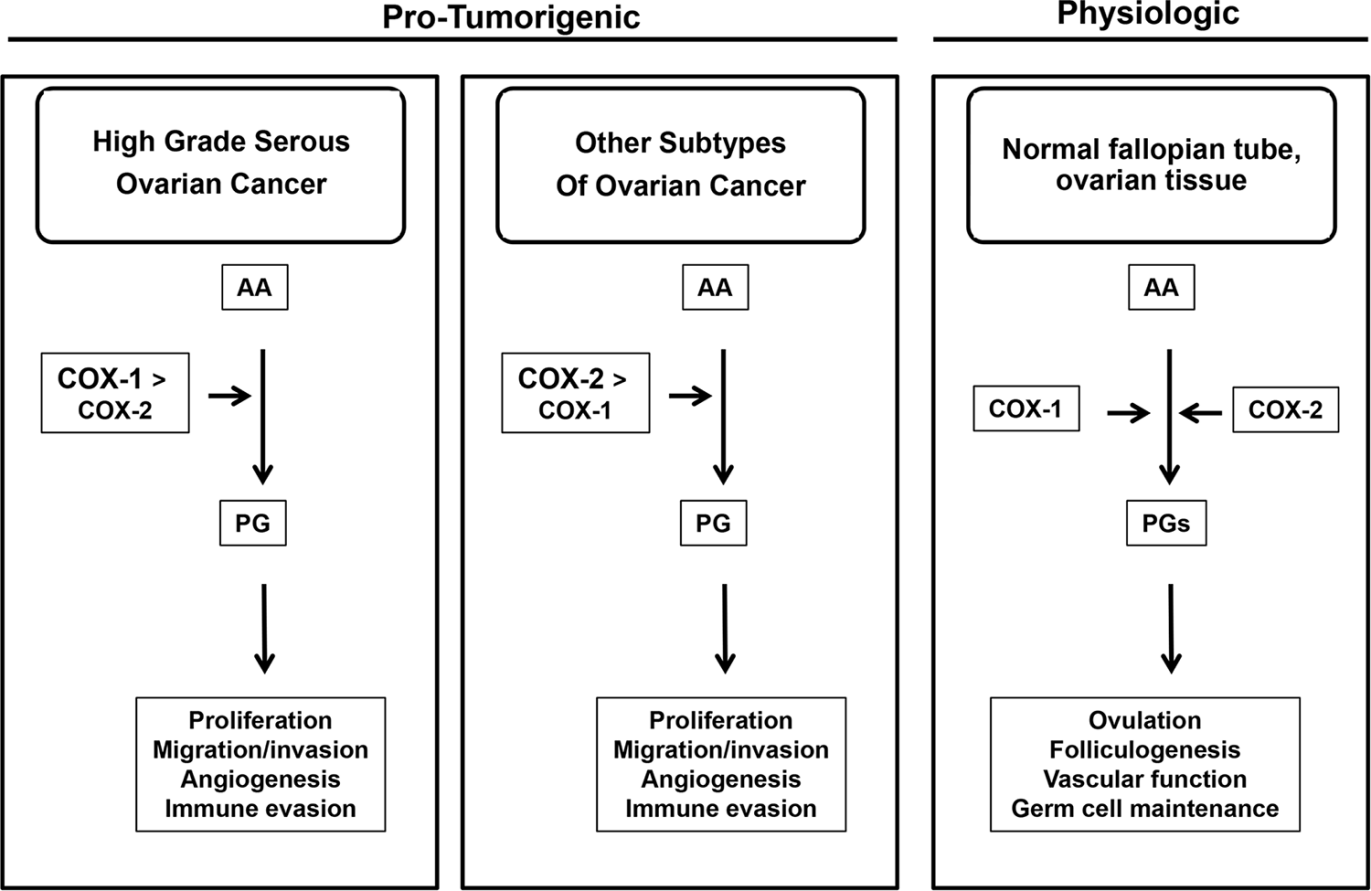
Conceptual Framework of COX-1 and COX-2 in Ovarian Cancer COX-1 appears to have an important pro-tumorigenic role in HGSOC tumors. In other histological subtypes of ovarian cancer, such as endometrioid and mucinous tumors, COX-2 may play a more dominant role. In normal ovarian tissue, COX-1 and/or COX-2 have important physiologic functions such as folliculogenesis and ovulation. The role of COX-1 in the normal fallopian tube is not clear.

Over 20 years ago, COX-1 was discovered as a tumor-associated antigen in ovarian cancer [3, 4]. Despite decades of research on COX-1 in ovarian cancer, conflicting and controversial preclinical and epidemiological results have limited the development of COX-1 as a molecular target. Our study was designed to address some of the controversies regarding the role of COX-1 in human ovarian cancer and to gain insight into specific tumor types that could benefit from COX-1 selective agents. Based on our results, we have developed the following conceptual framework for COX-1 in ovarian cancer (Figure 7). COX-1 appears to have an important pro-tumorigenic role in HGSOC tumors, the most biologically aggressive histological type of ovarian cancer, likely through PG signaling. In other histological subtypes of ovarian cancer, such as endometrioid and mucinous tumors, COX-2 may play a more dominant role. In normal ovarian tissue, COX-1 and/or COX-2 have important physiologic functions in folliculogenesis and ovulation [63–65]. Although the role of COX-1 in the fallopian tube is not clear, it could provide clues into the mechanisms of COX-1 overexpression in HGSOC tumors since the fallopian tube is a putative precursor for HGSOC tumors [66]. In conclusion, our study provides strong support for a role for COX-1 in HGSOC and continued development of COX-1 as a viable molecular target for diagnostic, chemopreventive, and therapeutic purposes in HGSOC tumors, the most common and aggressive type of ovarian cancer.

## MATERIALS AND METHODS

### Analysis of COX-1 and COX-2 expression in ovarian tumors

First, we used data extracted from The Cancer Genome Atlas (TCGA) project [27]. We obtained RNA-seq V2 data normalized by RSEM (RNA-seq by expectation maximization) software for *COX-1/PTGS1* and *COX-2/PTGS2* transcripts in HGSOC via the cBioPortal resource (http://www.cbioportal.org). TCGA ovarian cancer studies focused solely on HGSOC tumors. For the PANCAN tumor panel: bladder urothelial carcinoma (BLCA), breast invasive carcinoma (BRCA), colorectal adenocarcinoma (COAD), glioblastoma multiforme (GBM), head and neck squamous cell carcinoma (HNSC), kidney renal clear cell carcinoma (KIRC), acute myeloid leukemia (LAML), lung adenocarcinoma (LUAD), lung squamous cell carcinoma (LUSC), ovarian serous adenocarcinoma (OV) and uterine corpus endometrioid carcinoma (UCEC).

To assess basal *COX-1* and *COX-2* gene expression in ovarian cancer cell lines, we accessed the CellMiner resource Affymetrix U133 Plus 2.0 microarray data from 7 ovarian cancer cell lines in the NCI 60 cancer cell line panel [67] and the Broad-Novartis Institute Cancer Cell Line Encyclopedia (CCLE) for *COX-1* and *COX-2* Affymetrix U133 Plus 2.0 microarray mRNA expression data from 47 unique ovarian cancer cell lines [28]. Expression of *COX-1* and *COX-2* was compared to the corresponding “HGSOC similarity score” for each of the ovarian cancer cell lines, which was generated by comparing copy number changes, mutations and mRNA expression profiles to that characteristic of patient HGSOC tumors [29].

Finally, we used a fully annotated tissue microarray (TMA) generated in our laboratory from ovarian cancer patient serous, endometrioid, mucinous, and clear cell tumors [30] (Supplementary Table S3). Institutional Review Board approval for the tissue studies was obtained at Vanderbilt University Medical Center as previously published [30].

### Cell culture

Growth of the human epithelial ovarian cancer cell lines SKOV-3, OVCAR-3 and OVCAR-4, well-characterized as part of the National Cancer Institute (NCI) 60 Cancer Panel [67, 68], have been described previously [30]. The growth of SKOV-3 cells stably transfected with a pcDNA-COX1 expression vector (SK/COX1) and control cells stably expressing the empty vector (SK/PCDNA) has been described previously [30]. In addition, the following mouse ovarian cancer cell lines were used: ID8 [31] and genetically engineered mouse T1, T2 T2+RAS, T3 and TBR5 cells [32, 33]. Cell lines were authenticated in 2014 by the Vanderbilt Technologies for Advanced Genomics (VANTAGE) Core using the GenePrint 10 kit (Promega, Madison, WI). Gene loci profiles were verified using, where applicable, NCI 60, COGCELL, and ATCC reference databases. All cell lines used tested negative for Mycoplasma.

### Generation of COX-1 stable knockdown cells

OVCAR-3 cells were transfected (Lipofectamine 2000, Invitrogen Corp., Carlsbad, CA) with a pre-designed pRS shRNA HuSH-29 plasmid targeting human COX-1 or control, scrambled shRNA (ShScr) on the same vector background (Origene, Rockville, MD). Stably expressing OVCAR-3 clones were selected with 0.1 μg/ml puromycin (Sigma Chemical Co., St Louis, MO), and screened for COX-1 expression by western blot. Two independent clones displaying robust down-regulation of COX-1 protein levels (OV3/COX1KD) compared to clones expressing scrambled ShRNA (OV3/SCR) were selected for further analysis. COX-1 mRNA and protein knockdown was verified by quantitative real time RT-PCR and RNA-seq, and Western blot, respectively.

### Transient COX-1 knockdown

For transient knockdown of COX-1, cells were transfected with ON-TARGETplus non-targeting (NT) or COX-1-targeting siRNA duplexes (Thermo Fisher Scientific, Inc., Waltham, MA) using RNAiMAX transfection reagent (Invitrogen Corp.). Two independent combinations of duplexes were used to efficiently down-regulate COX-1 expression. Validation of COX-1 protein knockdown was performed by western blot analysis.

### RNA isolation and RNA-seq analysis

OV3/SCR and OV3/COX1KD cells were harvested in growing log phase and RNA isolation performed as previously described [69]. Three independent passages of cells were assayed by RNA-seq analysis of gene expression in our isogenic cell line pair of COX-1 proficiency and deficiency was performed by the Vanderbilt Technologies for Advanced Genomics Core (VANTAGE) core using the Illumina HiSeq2500 platform. RNA-seq reads were aligned to the human genome hg19 using TopHat2 [70] and the number of reads mapped to each gene was calculated by HTseq (http://www-huber.embl.de/users/anders/HTSeq/). Differentially expressed genes between the OV3/COX1KD and OV3/SCR cells were detected by DESeq2 [71]. The *P* values were corrected for multiple testing using the Benjamini-Hochberg procedure [72]. The significantly changed genes were determined based on fold change greater than 2.5 (FC>2.5) and the corrected *P* value less than 0.01 (FDR < 0.01). Functional enrichment analysis on differentially expressed genes to infer pathways and regulatory mechanisms associated with COX-1 expression was performed by WebGestalt (Web-based gene set analysis toolkit; http://bioinfo.vanderbilt.edu/webgestalt/) [73]. Enrichment p-values were generated by the Fisher's exact test and adjusted by Benjamini and Hochberg's multiple-test [72]. Raw and processed RNA-seq data files are available at the GEO repository http://www.ncbi.nlm.nih.gov/geo/query/acc.cgi?&acc=GSE66173.

### Quantitative real time RT-PCR (QPCR)

QPCR analysis was performed to compare mRNA expression of selected genes in OV3/SCR and OV3/COX1KD cells. In addition, three independent passages of SK/PCDNA and SK/COX1 cells were harvested in growing log phase and processed for RNA isolation. Levels of mRNA expression for *COX-1, COX-2*, and other established markers of cell proliferation, angiogenesis, epithelial-mesenchymal transition (EMT) and cell migration/invasion were determined using QPCR at the Vanderbilt VANTAGE Core. Taqman® probes for the following genes are in Supplementary Table S4: *COX-1, COX-2, CDKN1A* (p21), *CCND1* (cyclin D1), *VEGFC* (Vascular endothelial growth factor C), *CHD2* (N-cadherin), *SNAI1* (SNAIL), *MMP7* (matrix metalloproteinase 7), *MMP2, FN1* (fibronectin), *CHD1* (E-cadherin) and *GAPDH*. Additional details are provided in Supplementary Methods.

### Immunohistochemistry

Tissue fixation, processing and sectioning methods have been previously described [74]. Hematoxylin and eosin staining for histology and immunostaining for human COX-1, COX-2, PAX8 and p53 were performed as described [74]. Additional details for these antibodies are provided in Supplementary Materials and Methods. For our ovarian cancer TMAs, semi-quantitative measurement of COX-1 and COX-2 expression in tumors was performed using the automated Ariol® SL-50 Platform (Molecular Devices LLC, Sunnyvale, CA) in the Digital Histology Shared Resource (DHSR) at Vanderbilt University Medical Center (VUMC) (http://www.mc.vanderbilt.edu/dhsr). [30]. Staining of COX-1 and COX-2 for each tumor was classified as high (>50% positive tumor cells), moderate (10–50% positive tumor cells) or weak (~10% positive tumor cells), based on a meta-analysis of COX staining in ovarian cancer [21].

### Western blot analysis

Whole cell protein isolation, preparation of nuclear extracts, Western blotting and signal detection were performed, as described previously [30], to detect protein levels of COX-1, COX-2, VEGF, MMP2, cyclin D1, p21, E-cadherin, phospho-Smad 1/5, p65/RelA and RelB. Corresponding levels of β-actin and histone H3 were used as loading controls for whole and nuclear extracts, respectively. Additional details are provided in Supplementary Materials and Methods.

### Functional cellular assays

Cellular COX activity was measured by stimulation with 50 μM ^14^C-arachidonic acid (30 s at 37°C) followed by quantification of radiolabeled prostaglandin products, as described [62]. The percentage of total products was expressed per 10^6^ cells. Cellular secretion of VEGF into serum-free medium with and without stimulation with arachidonic acid (20 μM) for 24 hours was measured using a human VEGF ELISA plate according to manufacturer's instructions (Signosis Inc, Santa Clara, CA). Sulfurhodamine B (SRB) growth assays were performed as previously described [31, 69]. Effects on cell growth were measured 72 hours after plating of cells. Clonogenic assays were performed and quantified using the GelCount System (Oxford Optronix, UK) in the DHSR at VUMC [69]. Cell invasion assays were performed using low basement membrane cell invasion inserts according to manufacturer's instructions (Trevigen, Gaithersburg, MD). Standard 10% FBS growth medium was used as a chemoattractant in the lower chamber for cells grown on the inserts. Following invasion, cells on the side of the insert facing the lower chamber were stained with 1% crystal violet, and the insert carefully mounted on a microscope slide for quantification of invading cells. 5 fields per insert were counted using a 5x objective lens.

### Statistical analysis

Differences between COX-1 and COX-2 mRNA and protein levels in ovarian tumors, and differences in COX-1 expression between ovarian TCGA tumors and the remaining tumor histiotypes in the PANCAN tumor panel, were determined by Mann-Whitney test, with *p* < 0.05 considered to be statistically significant. For comparisons of stratified TMA COX-1 and COX-2 staining (high, moderate, or weak) with respect to tumor histological subtypes and serous tumor grade, a chi-squared test was employed. When expected cell counts were less than 5, the Fisher's exact test was employed. For the *in vitro* experiments, values shown were based on the mean + SD of 3 independent experiments. Differences relative to appropriate controls were evaluated by the Student's *t*-test, with *p* < 0.05 considered to be statistically significant.

## SUPPLEMENTARY METHODS


