# Supplementary material for: Aberrant over-expression of COX-1 intersects multiple pro-tumorigenic pathways in high-grade serous ovarian cancer
Source: Oncotarget. 2015 May 4;6(25):21353–68. doi: 10.18632/oncotarget.3860 (PMC4673270; doi:10.18632/oncotarget.3860)
Supplement: Supplementary file 1 [file oncotarget-06-21353-s001.pdf]

SUPPLEMENTARY METHODS

Western blotting

The following primary antibodies were used: mouse monoclonal anti-COX-1 (Santa Cruz Biotechnology, Dallas, TX) (1:1000 dilution), rabbit polyclonal anti-COX-2 (Cayman Chemicals, Ann Arbor, MI.) (1:500 dilution), rabbit polyclonal anti-MMP2 (Abcam, Cambridge, MA) (1:1000 dilution), rabbit polyclonal anti-VEGF (Abcam) (1:1000 dilution), rabbit polyclonal anti-cyclin D1 (Abcam, Cambridge, MA) (1:500 dilution), rabbit polyclonal anti-Ecadherin (Cell Signaling Technology, Beverly, MA) (1:500 dilution), mouse monoclonal antip21 (Neomarkers, Fremont, CA) (1:250 dilution), rabbit polyclonal anti-phospho-Smad 1/5 (Cell Signaling Technology) (1:250 dilution), rabbit polyclonal anti-p65/RelA (Cell Signaling Technology) (1:1000 dilution), rabbit polyclonal anti-RelB (Cell Signaling Technology) (1:500 dilution), mouse monoclonal anti-β-Actin (Sigma Chemical Co.) (1:10000 dilution) and mouse monoclonal anti-histone H3 (EMD Millipore, Billerica, MA).

Quantitative real time RT-PCR

Total RNA was isolated using the Qiagen RNeasy Kit (Qiagen, Valencia, CA) according to manufacturer's instructions. Levels of mRNA expression were determined using Life Technologies (Grand Island, NY) FAM-MBG TaqMan® gene expression assays using the 7900HT real-time PCR machine (Applied Biosystems, Foster City, CA). Genes examined and their respective TaqMan® probes are listed in Supplementary Table S4. Relative expression was measured compared to an internal GAPDH standard, and calculated using the $2^{-\Delta\Delta CT}$ method.

Immunohistochemistry

In human ovarian tumor tissue, immunostaining for the following primary antibodies was performed in the Vanderbilt Immunohistochemistry Core Facility: mouse polyclonal anti-COX-1 (Santa Cruz Biotechnology) (1:100 dilution for 1 hour); mouse monoclonal anti-COX-2 (ThermoFisher Scientific) (1:300 dilution for 1 hour); rabbit polyclonal anti-PAX8 (Proteintech, Chicago, IL,(1:50 for 1 hour) and p53 (Ready-To-Use, #PA0057, Leica-Microsystems, Buffalo Grove, IL).

SUPPLEMENTARY FIGURES AND TABLES

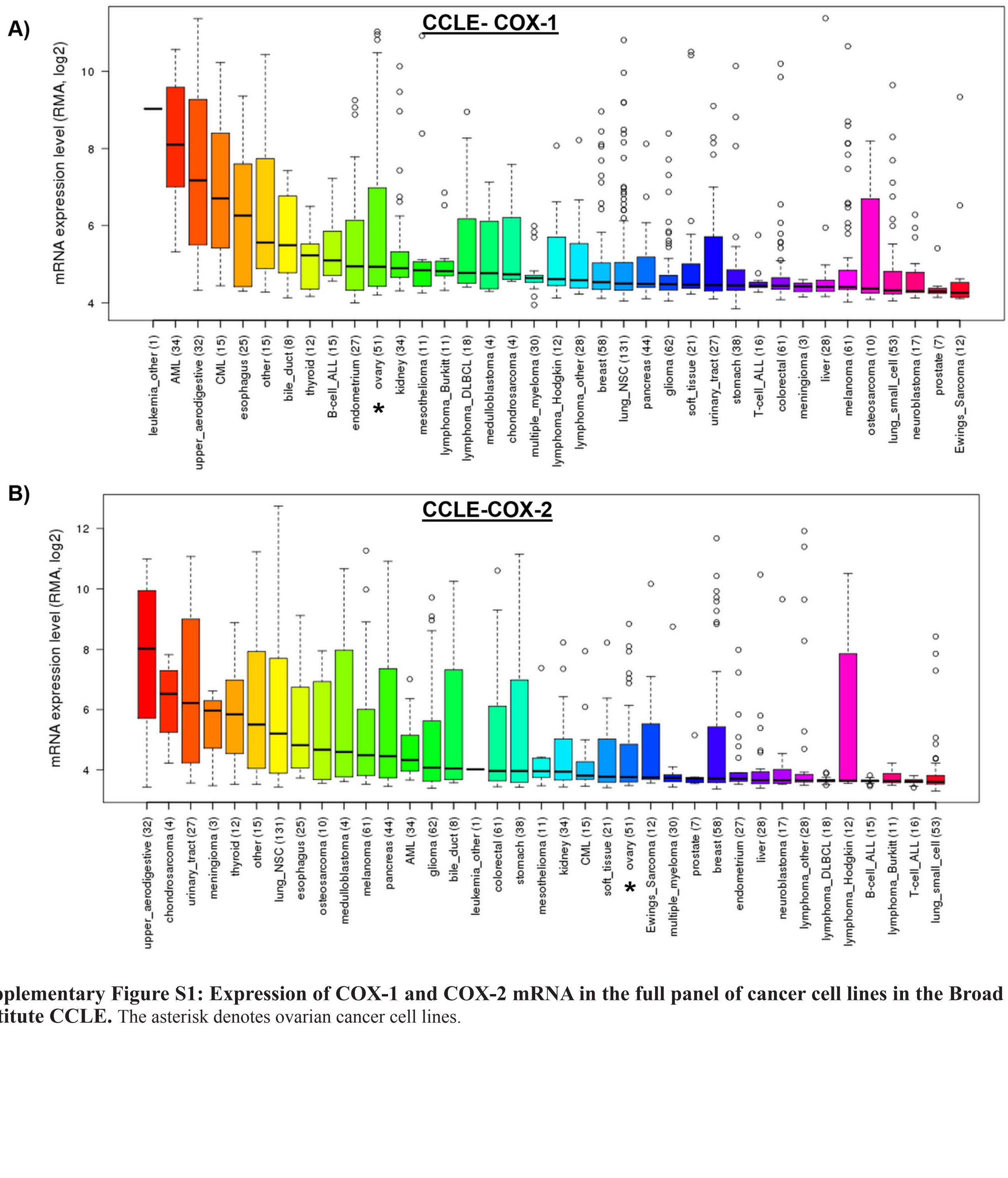

Supplementary Figure S1: Expression of COX-1 and COX-2 mRNA in the full panel of cancer cell lines in the Broad Institute CCLE. The asterisk denotes ovarian cancer cell lines.

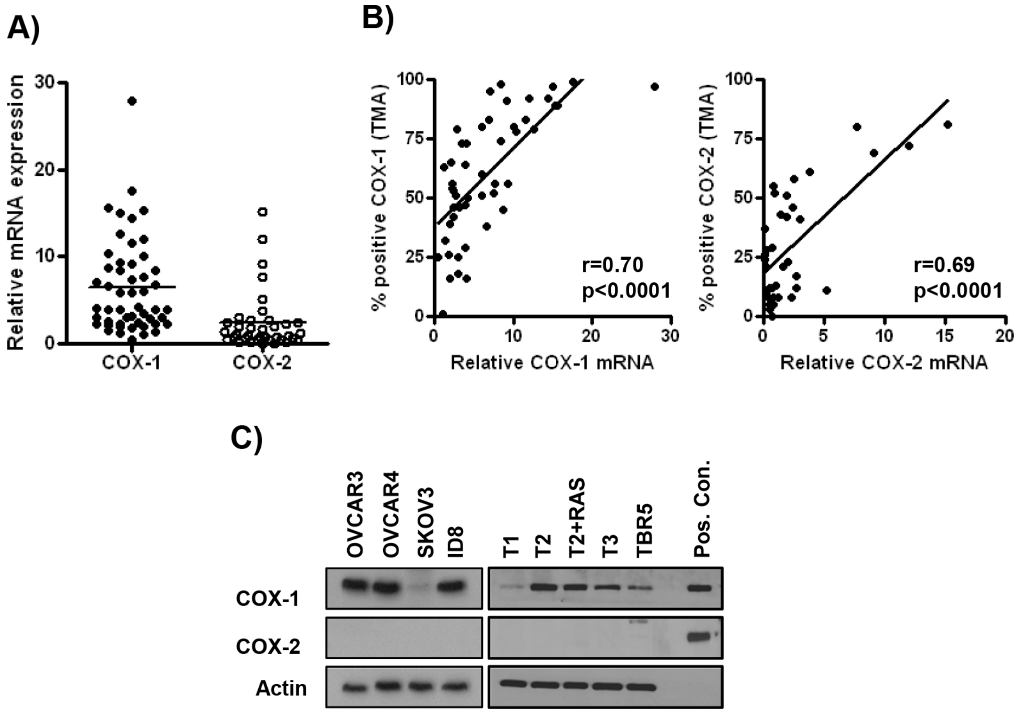

Supplementary Figure S2: A. QPCR analysis of COX-2 mRNA expression in a subset of serous tumors from the Khabele TMA. **B.** Spearman correlation of COX-1 protein and mRNA expression in a subset of 52 serous tumors in the TMA. **C.** Western blot analysis of protein expression of COX-1 and COX-2 in human and mouse-derived ovarian cancer cell lines. Actin was the loading control.

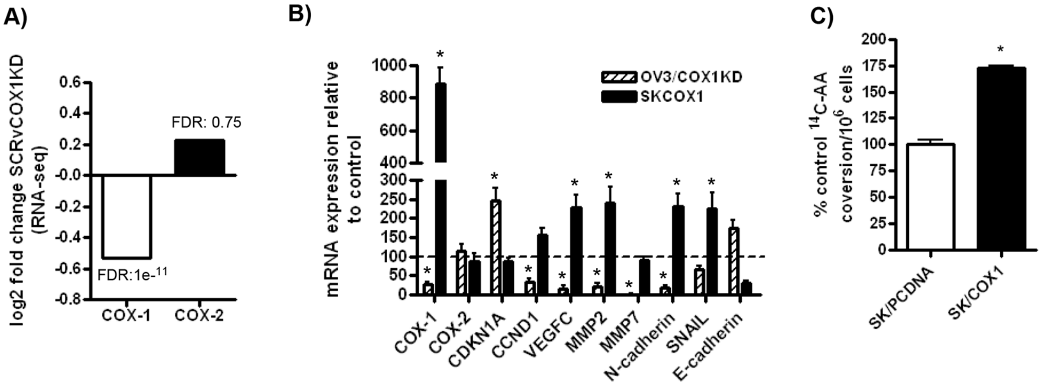

Supplementary Figure S3: A. RNA-seq analysis of COX-1 and COX-2 mRNA expression in OV3/COX1KD clone #2 compared to OV3/SCR clone #1 cells. FDR values are shown for DESeq2 analyses. Values are log2 fold change for 3 replicate experiments. **B.** Effects of COX-1 down-regulation on mRNA expression of selected genes were confirmed in QPCR assays. Effects of COX-1 over-expression in SK/COX1 cells were also determined. Values are expressed as a percentage of the respective control cell expression. **C.** Conversion of 14C arachidonic acid (14C-AA) to prostaglandin products in SK/PCDNA and SK/COX1 cells after 30 min stimulation in serum-free medium. Results are expressed as a percentage of conversion in control SK/PCDNA cells per 106 cells. Values are mean + SEM of least 2 independent experiments. *$p < 0.01$ compared to corresponding control cell line, Student's *t* test.

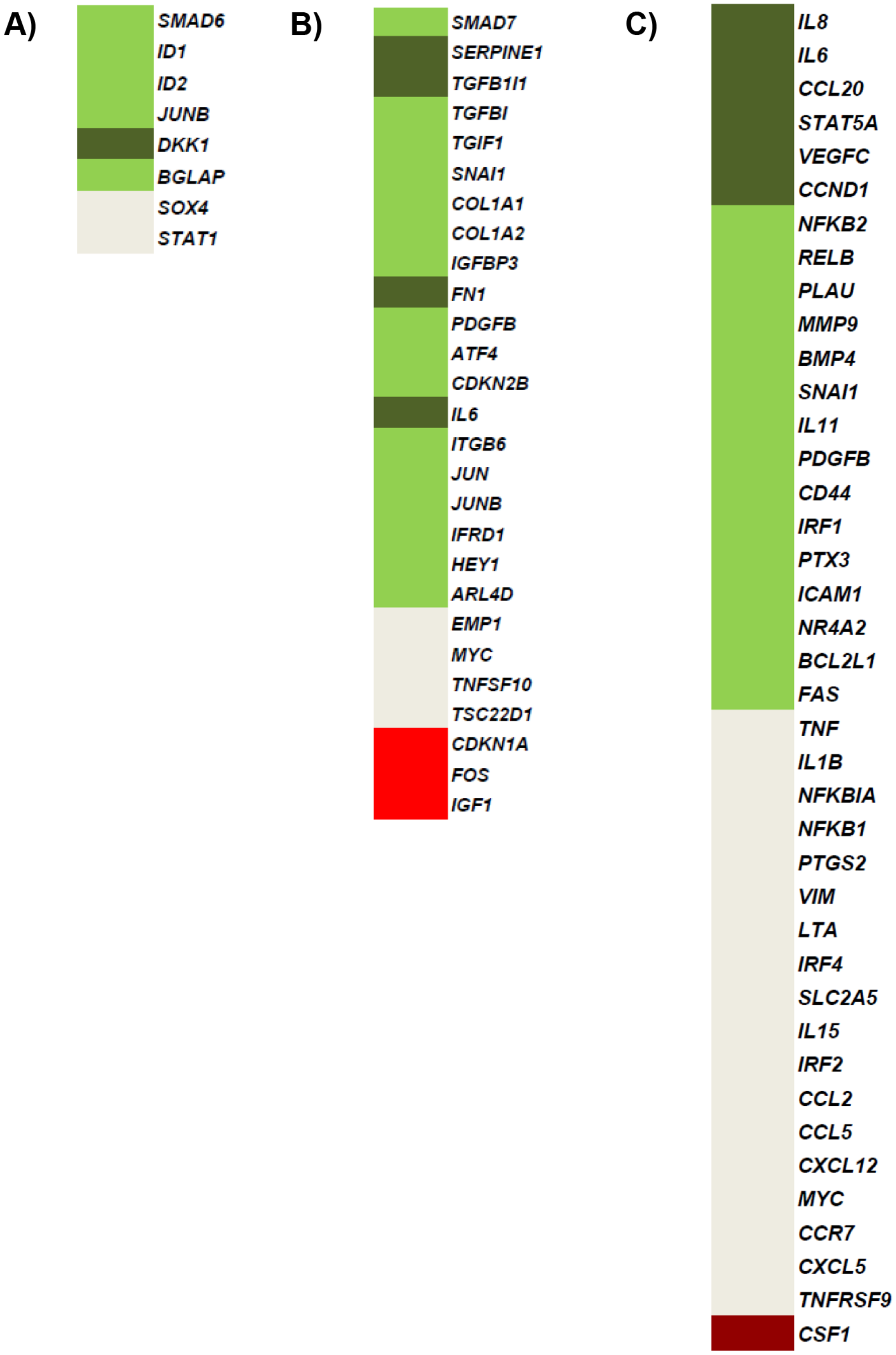

Supplementary Figure S4: Expression of established targets of A. BMP, B. TGF-β and C. NF-κB pathway signaling in OV3/COX1KD cells compared to control OV3/SCR cells in RNA-seq experiments. Light green (log2 fold change < –0.5, FDR < 0.01), dark green (log2 fold change<−1.5, FDR < 0.01), light red (log2 fold change>0.5, FDR < 0.01), dark red (log2 fold change > 1.5, FDR < 0.01), DeSeq2 analysis. NF-κB target were assessed using the Boston University Biology resource http://www.bu.edu/nf-kb/gene-resources/target-genes/**.**

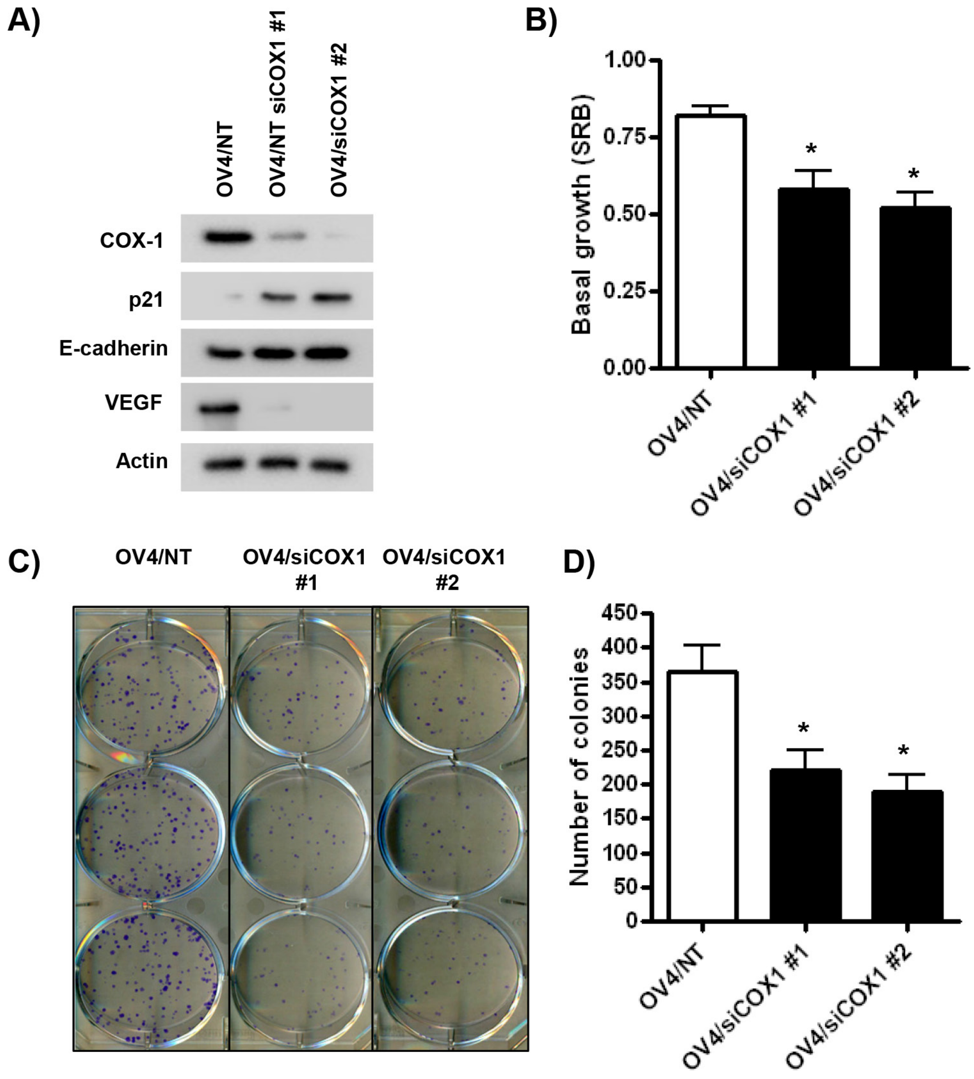

Supplementary Figure S5: A. Relative effects of COX-1 down-regulation in OVCAR-4 cells on protein expression of selected genes were confirmed by western blot. Actin was used as loading control. Two independent combinations of COX-1 targeting siRNA (siCOX1) were assayed compared to the effects of control, non-targeting siRNA (NT) (all 100 nM). **B.** SRB assay (72 h) and **C–D.** clonogenic assay in OVCAR-4 cells with (OV4/COX1KD) and without (OV4/NT) selective COX-1 down-regulation. 500 cells were seeded per cell line treatment and colonies allowed to grow for 10 days. Values are mean + SEM of 3 independent experiments. *$p < 0.01$ relative to NT-treated OVCAR-4 cells, Student's *t* test.

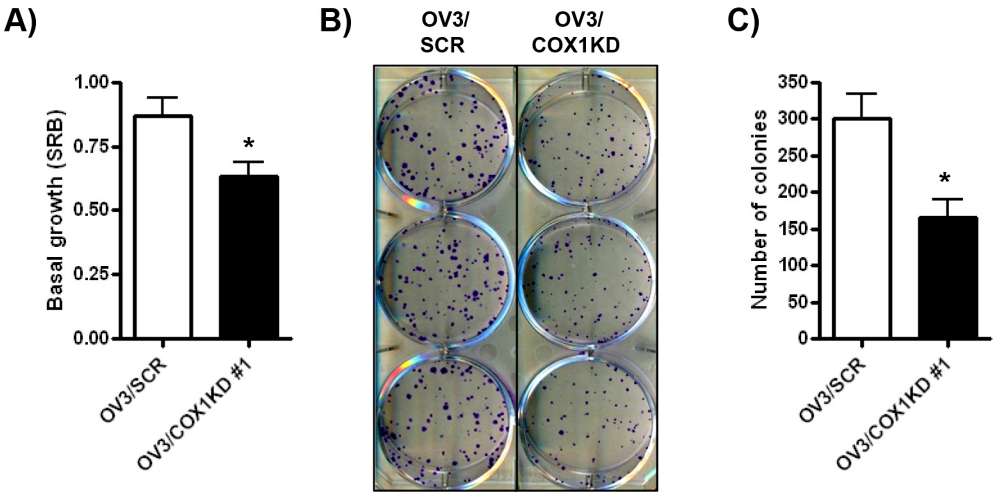

Supplementary Figure S6: A. SRB growth assay (72 h) and **B–C.** clonogenic assay of OV3/COX1KD clone #1 compared to OV3/SCR. For clonogenic assays, 500 cells were seeded per cell line and colonies allowed to grow for 14 days. Values are mean + SEM of 3 independent experiments *n*. $^*p < 0.01$, Student's *t* test.

Supplementary Table S1: Average transcript intensity COX-1 Z scores

Cell lines	Average z scores
BR:MCF7	–0.26
BR:MDA_MB_231	0.23
BR:HS578T	–0.12
BR:BT_549	–0.3
BR:T47D	–0.53
CNS:SF_268	–0.65
CNS:SF_295	–0.04
CNS:SF_539	–0.42
CNS:SNB_19	–0.4
CNS:SNB_75	–0.2
CNS:U251	–0.1
CO:COLO205	–0.31
CO:HCC_2998	–0.45
CO:HCT_116	0.25
CO:HCT_15	–0.22
CO:HT29	–0.3
CO:KM12	–0.26
CO:SW_620	–0.21
LE:CCRF_CEM	–0.47
LE:HL_60	1.57
LE:K_562	1.3
LE:MOLT_4	–0.29
LE:RPMI_8226	0.11
LE:SR	–0.61
ME:LOXIMVI	–0.48
ME:MALME_3M	–0.61
ME:M14	–0.3
ME:SK_MEL_2	–0.56
ME:SK_MEL_28	–0.3
ME:SK_MEL_5	–0.53
ME:UACC_257	–0.49
ME:UACC_62	–0.2
ME:MDA_MB_435	–0.46
ME:MDA_N	–0.24
LC:A549	–0.18

(*Continued*)

**LC:EKVX**	0.11
**LC:HOP_62**	0.23
**LC:HOP_92**	–0.17
**LC:NCI_H226**	–0.35
**LC:NCI_H23**	–0.58
**LC:NCI_H322M**	–0.44
**LC:NCI_H460**	0.24
**LC:NCI_H522**	–0.45
**OV:IGROV1**	–0.02
**OV:OVCAR_3**	2.34
**OV:OVCAR_4**	5.18
**OV:OVCAR_5**	0.48
**OV:OVCAR_8**	–0.41
**OV:SK_OV_3**	–0.18
**OV:NCI_ADR_RES**	–0.29
**PR:PC_3**	–0.47
**PR:DU_145**	–0.24
**RE:786_0**	0.41
**RE:A498**	0.09
**RE:ACHN**	0.1
**RE:CAKI_1**	0.36
**RE:RXF_393**	0.14
**RE:SN12C**	–0.21
**RE:TK_10**	0.26
**RE:UO_31**	0.54

Values are percentage of tumor cells staining positive for COX-1 or COX-2 by automated image analysis. NS = no spot. N/A represent tumors of non-epithelial origin.

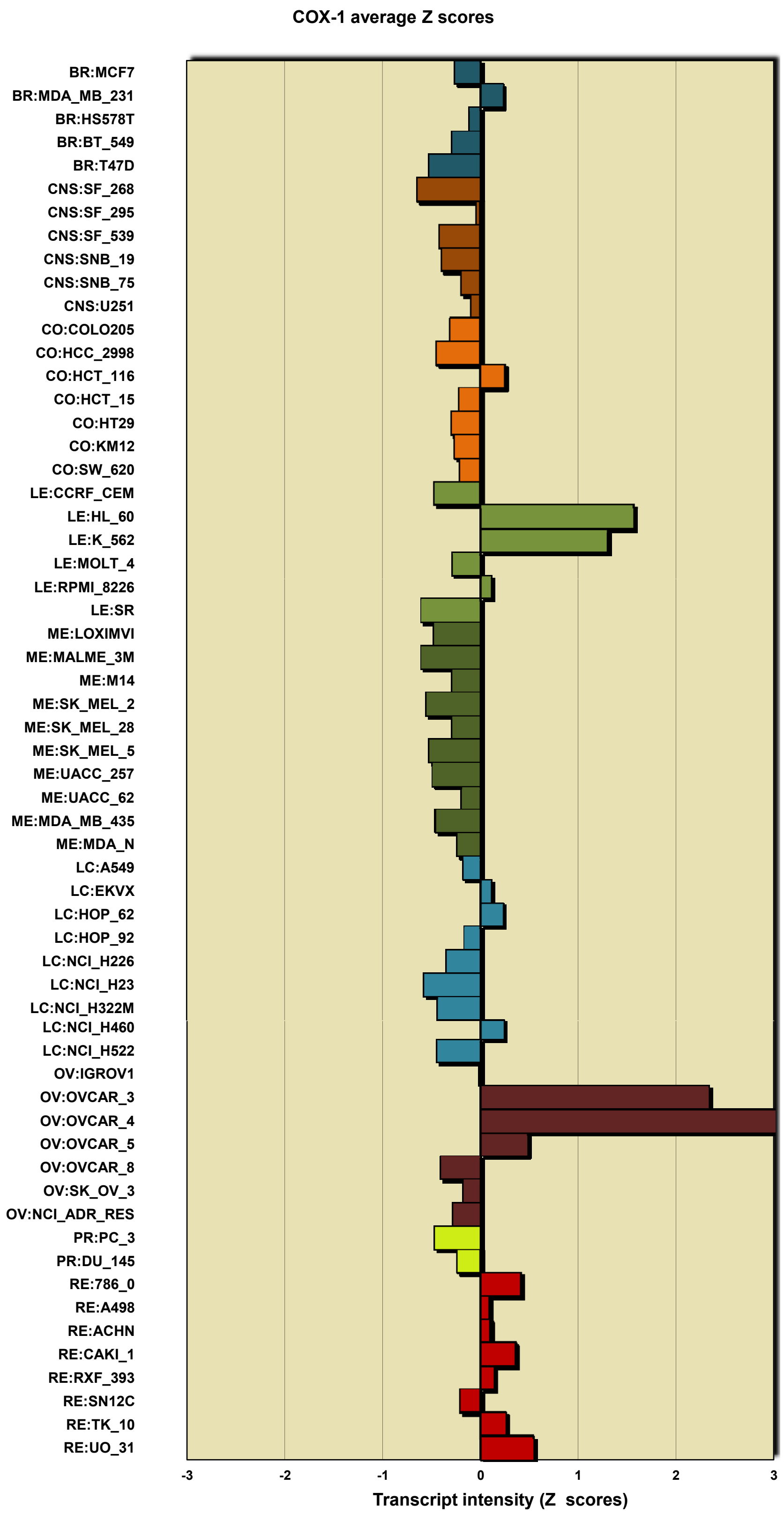
COX-1 average Z scores
BR:MCF7
BR:MDA_MB_231
BR:HS578T
BR:BT_549
BR:T47D
CNS:SF_268
CNS:SF_295
CNS:SF_539
CNS:SNB_19
CNS:SNB_75
CNS:U251
CO:COLO205
CO:HCC_2998
CO:HCT_116
CO:HCT_15
CO:HT29
CO:KM12
CO:SW_620
LE:CCRF_CEM
LE:HL_60
LE:K_562
LE:MOLT_4
LE:RPMI_8226
LE:SR
ME:LOXIMVI
ME:MALME_3M
ME:M14
ME:SK_MEL_2
ME:SK_MEL_28
ME:SK_MEL_5
ME:UACC_257
ME:UACC_62
ME:MDA_MB_435
ME:MDA_N
LC:A549
LC:EKVX
LC:HOP_62
LC:HOP_92
LC:NCI_H226
LC:NCI_H23
LC:NCI_H322M
LC:NCI_H460
LC:NCI_H522
OV:IGROV1
OV:OVCAR_3
OV:OVCAR_4
OV:OVCAR_5
OV:OVCAR_8
OV:SK_OV_3
OV:NCI_ADR_RES
PR:PC_3
PR:DU_145
RE:786_0
RE:A498
RE:ACHN
RE:CAKI_1
RE:RXF_393
RE:SN12C
RE:TK_10
RE:UO_31
-3
-2
-1
0
1
2
3
Transcript intensity (Z scores)

Supplementary Table S2: Average transcript intensity COX-2 Z scores

Cell lines	Average z scores
BR:MCF7	–0.4
BR:MDA_MB_231	–0.6
BR:HS578T	1.09
BR:BT_549	–0.43
BR:T47D	–0.5
CNS:SF_268	–0.6
CNS:SF_295	–0.43
CNS:SF_539	1.19
CNS:SNB_19	–0.68
CNS:SNB_75	–0.03
CNS:U251	–0.36
CO:COLO205	–0.59
CO:HCC_2998	–0.23
CO:HCT_116	–0.35
CO:HCT_15	–0.65
CO:HT29	0.51
CO:KM12	0.17
CO:SW_620	–0.6
LE:CCRF_CEM	–0.55
LE:HL_60	–0.26
LE:K_562	–0.5
LE:MOLT_4	–0.66
LE:RPMI_8226	–0.4
LE:SR	–0.76
ME:LOXIMVI	1.4
ME:MALME_3M	–0.54
ME:M14	0.9
ME:SK_MEL_2	–0.59
ME:SK_MEL_28	0.38
ME:SK_MEL_5	0.12
ME:UACC_257	–0.6
ME:UACC_62	0.65
ME:MDA_MB_435	–0.71
ME:MDA_N	–0.55
LC:A549	2.29
LC:EKVX	4.09

(*Continued*)

**LC:HOP_62**	0.89
**LC:HOP_92**	–0.43
**LC:NCI_H226**	–0.38
**LC:NCI_H23**	0.79
**LC:NCI_H322M**	2.14
**LC:NCI_H460**	1.32
**LC:NCI_H522**	–0.1
**OV:IGROV1**	–0.54
**OV:OVCAR_3**	–0.32
**OV:OVCAR_4**	–0.55
**OV:OVCAR_5**	–0.44
**OV:OVCAR_8**	–0.57
**OV:SK_OV_3**	–0.62
**OV:NCI_ADR_RES**	1.06
**PR:PC_3**	1.42
**PR:DU_145**	–0.62
**RE:786_0**	–0.63
**RE:A498**	–0.32
**RE:ACHN**	–0.41
**RE:CAKI_1**	–0.36
**RE:RXF_393**	0.38
**RE:SN12C**	–0.54
**RE:TK_10**	–0.13
**RE:UO_31**	–0.32

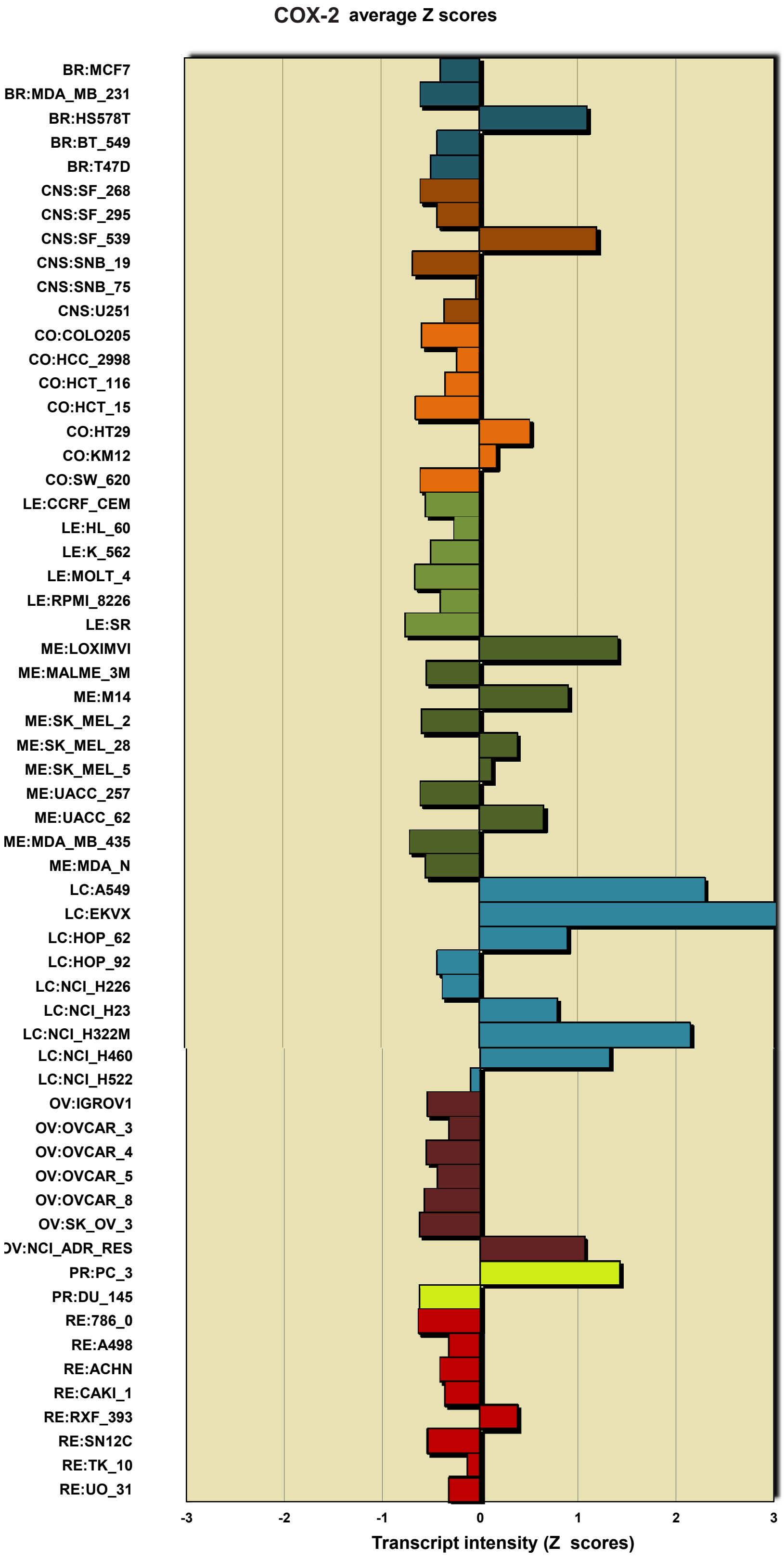
COX-2 average Z scores
BR:MCF7
BR:MDA_MB_231
BR:HS578T
BR:BT_549
BR:T47D
CNS:SF_268
CNS:SF_295
CNS:SF_539
CNS:SNB_19
CNS:SNB_75
CNS:U251
CO:COLO205
CO:HCC_2998
CO:HCT_116
CO:HCT_15
CO:HT29
CO:KM12
CO:SW_620
LE:CCRF_CEM
LE:HL_60
LE:K_562
LE:MOLT_4
LE:RPMI_8226
LE:SR
ME:LOXIMVI
ME:MALME_3M
ME:M14
ME:SK_MEL_2
ME:SK_MEL_28
ME:SK_MEL_5
ME:UACC_257
ME:UACC_62
ME:MDA_MB_435
ME:MDA_N
LC:A549
LC:EKVX
LC:HOP_62
LC:HOP_92
LC:NCI_H226
LC:NCI_H23
LC:NCI_H322M
LC:NCI_H460
LC:NCI_H522
OV:IGROV1
OV:OVCAR_3
OV:OVCAR_4
OV:OVCAR_5
OV:OVCAR_8
OV:SK_OV_3
OV:NCI_ADR_RES
PR:PC_3
PR:DU_145
RE:786_0
RE:A498
RE:ACHN
RE:CAKI_1
RE:RXF_393
RE:SN12C
RE:TK_10
RE:UO_31
-3
-2
-1
0
1
2
3
Transcript intensity (Z scores)

Supplementary Table S3: Protein expression of COX-1 and COX-2 and tumor characteristics in 209 ovarian cancer patients

Study ID	% COX1	% COX2	Tumor stage	Tumor grade	Epithelial type
VOC-A-001	**0.3**	**85.2**	IIC	Grade 3	mucinous
VOC-A-002	**91.8**	**72.3**	IIIC	Grade 2	serous/papillary
VOC-A-003	**37.6**	**57.9**	IIIC	Grade 3	serous/papillary
VOC-A-004	**25.6**	**4.8**	IIIC	Grade 3	serous/papillary
VOC-A-006	**55.8**	**8.3**	IIIC	Grade 3	serous/papillary
VOC-A-007	**50.1**	**31.4**	IIIC	Grade 3	serous/papillary
VOC-A-008	**42.0**	**7.9**	IIIC	Grade 2	serous/papillary
VOC-A-009	**64.6**	**40.7**	IIIC	Grade 3	serous/papillary
VOC-A-012	**64.1**	**12.2**	IIIC	Grade 2	serous/papillary
VOC-A-013	**50.8**	**42.8**	IIIC	Grade 3	serous/papillary
VOC-A-014	**95.3**	**68.9**	IIIC	Grade 1	serous/papillary
VOC-A-015	**38.9**	**24.1**	IIIC	Grade 3	serous/papillary
VOC-A-016	**45.7**	**13.0**	IIIC	Grade 3	serous/papillary
VOC-A-017	**0.4**	**98.3**	IIIC	Grade 2	endometrioid
VOC-A-018	**3.8**	**2.3**	IIIC	Grade 3	clear cell
VOC-A-020	**0.1**	**29.9**	IA	Grade 2	N/A
VOC-A-024	**0.0**	**0.3**	IC	Grade 1	N/A
VOC-A-025	**2.9**	**34.0**	unstaged	Grade 1	mucinous
VOC-A-026	**80.1**	**36.8**	IV	Grade 3	serous/papillary
VOC-A-027	**73.0**	**29.2**	IIIC	Grade 2	serous/papillary
VOC-A-028	**54.4**	**60.8**	IV	Grade 3	serous/papillary
VOC-A-030	**89.3**	**64.2**	IIIC	Grade 3	serous/papillary
VOC-A-032	**NS**	**NS**	IIIC	Grade 3	serous/papillary
VOC-A-033	**52.1**	**4.7**	IIIC	Grade 3	serous/papillary
VOC-A-034	**73.4**	**10.5**	IIIC	Grade 3	serous/papillary
VOC-A-036	**NS**	**NS**	unstaged	Grade 3	serous/papillary
VOC-A-037	**55.5**	**22.9**	IIIC	Grade 2	serous/papillary
VOC-A-038	**77.6**	**28.0**	IIIC	Grade 3	serous/papillary
VOC-A-039	**97.7**	**24.0**	IIIC	Grade 3	serous/papillary
VOC-A-040	**62,5**	**21.3**	IIIC	Grade 3	serous/papillary
VOC-A-041	**0.3**	**98.2**	IA	Grade 1	endometrioid
VOC-A-042	**NS**	**NS**	IC	Grade 1	endometrioid
VOC-A-043	**75.1**	**80.5**	IA	Grade 3	clear cell
VOC-A-044	**66.9**	**70.5**	IIIC	Grade 3	mixed
VOC-A-045	**NS**	**NS**	IIIB	Grade 3	N/A
VOC-A-047	**73.8**	**21.1**	IIIC	Grade 3	serous/papillary

(*Continued*)

VOC-A-048	**61.6**	**4.8**	IIIC	Grade 3	N/A
VOC-A-052	**0.0**	**64.8**	IA	Grade 3	mucinous
VOC-A-053	**56.3**	**5.7**	IIIC	Grade 3	serous/papillary
VOC-A-055	**24.7**	**8.0**	IIC	Grade 2	serous/papillary
VOC-A-056	**25.0**	**21.8**	IIIC	Grade 3	serous/papillary
VOC-A-057	**92.2**	**1.8**	IV	Grade 3	serous/papillary
VOC-A-058	**44.9**	**11.0**	IIC	Grade 3	serous/papillary
VOC-A-059	**96.5**	**51.0**	IIIC	Grade 3	serous/papillary
VOC-A-060	**82.7**	**26.0**	IIIC	Grade 3	serous/papillary
VOC-A-061	**28.5**	**8.2**	IV	Grade 2	serous/papillary
VOC-A-063	**99.2**	**20.5**	IIIB	Grade 2	serous/papillary
VOC-A-064	**46.0**	**11.2**	IV	Grade 2	serous/papillary
VOC-A-065	**31.7**	**45.6**	IIIC	Grade 2	serous/papillary
VOC-A-066	**80.9**	**4.9**	IV	Grade 2	mixed
VOC-A-067	**17.6**	**1.8**	IIIC	Grade 3	serous/papillary
VOC-A-070	**83.0**	**11.9**	IV	Grade 3	serous/papillary
VOC-A-071	**79.7**	**54.8**	IIIB	Grade 3	serous/papillary
VOC-A-072	**47.0**	**11.5**	IIIC	Grade 2	serous/papillary
VOC-A-074	**68.2**	**48.7**	IA	Grade 3	other
VOC-A-075	**0.4**	**80.2**	unstaged	Grade 2	serous/papillary
VOC-A-076	**56.3**	**21.9**	IIIC	Grade 3	serous/papillary
VOC-A-078	**45.1**	**32.8**	IC	Grade 2	endometrioid
VOC-A-080	**0.2**	**4.9**	IA	Grade 3	clear cell
VOC-A-083	**0.1**	**10.4**	IA	Grade 3	clear cell
VOC-A-084	**4.6**	**5.4**	IA	Grade 3	other
VOC-A-085	**0.1**	**99.4**	IA	Grade 1	mucinous
VOC-A-086	**84.6**	**53.0**	IB	Grade 1	serous/papillary
VOC-A-088	**54.0**	**11.2**	IIIC	Grade 3	N/A
VOC-A-090	**51.0**	**41.9**	IIIC	Grade 3	serous/papillary
VOC-A-091	**88.6**	**51.5**	IIIC	Grade 3	serous/papillary
VOC-A-093	**97.2**	**25.5**	IIC	Grade 3	serous/papillary
VOC-A-094	**20.5**	**17.9**	IIIC	Grade 3	serous/papillary
VOC-A-095	**52.8**	**11.0**	IIIC	Grade 3	serous/papillary
VOC-A-096	**16.3**	**0.2**	IIIC	Grade 3	serous/papillary
VOC-A-098	**78.5**	**16.8**	IC	Grade 3	serous/papillary
VOC-A-099	**79.2**	**80.5**	IIIB	Grade 1	serous/papillary
VOC-A-101	**16.1**	**2.9**	IIIC	Grade 2	serous/papillary
VOC-A-102	**78.5**	**19.3**	IV	Grade 3	endometrioid

(*Continued*)

VOC-A-103	**0.2**	**72.0**	IA	Grade 3	endometrioid
VOC-A-104	**1.8**	**36.6**	IA	Grade 1	endometrioid
VOC-A-105	**0.0**	**50.4**	IA	Grade 2	endometrioid
VOC-A-106	**1.8**	**39.1**	IC	Grade 3	clear cell
VOC-A-107	**90.6**	**16.8**	IV	Grade 3	serous/papillary
VOC-A-109	**45.5**	**2.9**	IIIC	Grade 3	mixed
VOC-A-139	**3.7**	**6.9**	IV	Grade 3	endometrioid
VOC-A-141	**0.4**	**33.8**	IA	Grade 1	endometrioid
VOC-A-142	**17.0**	**58.3**	IIIC	Grade 3	endometrioid
VOC-A-145	**0.2**	**0.4**	IA	Grade 3	clear cell
VOC-A-150	**0.2**	**95.6**	IA	Grade 1	mucinous
VOC-A-152	**0.1**	**76.3**	IC	Grade 1	mucinous
VOC-A-153	**0.0**	**55.9**	IA	Grade 1	mucinous
VOC-A-154	**NS**	**NS**	IIIC	Grade 3	mucinous
VOC-A-155	**91.9**	**59.2**	IIIC	Grade 3	serous/papillary
VOC-A-156	**11.9**	**6.1**	IV	Grade 3	serous/papillary
VOC-A-157	**64.8**	**5.4**	IIIC	Grade 3	serous/papillary
VOC-A-158	**42.0**	**8.1**	IIIC	Grade 3	serous/papillary
VOC-A-159	**22.5**	**43.1**	IIIC	Grade 3	serous/papillary
VOC-A-160	**10.1**	**5.9**	IIIC	Grade 2	serous/papillary
VOC-A-161	**27.1**	**3.7**	IIIC	Grade 3	serous/papillary
VOC-A-162	**60.4**	**19.8**	IC	Grade 3	serous/papillary
VOC-A-163	**57.7**	**14.9**	IIIC	Grade 3	serous/papillary
VOC-A-164	**98.1**	**29.5**	IIIC	Grade 3	serous/papillary
VOC-A-165	**94.8**	**70.1**	IA	Grade 1	serous/papillary
VOC-A-166	**91.7**	**52.0**	IA	Grade 1	serous/papillary
VOC-A-167	**19.9**	**80.2**	IIIC	Grade 3	endometrioid
VOC-A-170	**1.9**	**71.8**	IC	Grade 2	endometrioid
VOC-A-171	**44.5**	**1.9**	IIIC	Grade 3	endometrioid
VOC-A-173	**NS**	**NS**	IA	Grade 2	endometrioid
VOC-A-174	**24.9**	**0.2**	IC	Grade 3	clear cell
VOC-A-178	**4.9**	**99.8**	IA	Grade 1	mucinous
VOC-A-179	**0.0**	**60.4**	IIIB	Grade 1	mucinous
VOC-A-180	**83.8**	**10.2**	IC	Grade 3	serous/papillary
VOC-A-181	**24.1**	**58.7**	IA	Grade 2	serous/papillary
VOC-A-182	**31.3**	**1.7**	IIIC	Grade 3	serous/papillary
VOC-A-183	**48.0**	**28.9**	IIIC	Grade 3	serous/papillary
VOC-A-184	**90.9**	**8.3**	IV	Grade 3	serous/papillary

(*Continued*)

VOC-A-185	**89.3**	**27.8**	IC	Grade 2	serous/papillary
VOC-A-188	**71.8**	**14.6**	IV	Grade 3	serous/papillary
VOC-A-189	**54.0**	**5.2**	IIIC	Grade 3	serous/papillary
VOC-A-190	**13.6**	**4.1**	IIIC	Grade 3	serous/papillary
VOC-A-191	**50.9**	**1.6**	IIIC	Grade 2	serous/papillary
VOC-A-192	**NS**	**NS**	IIIC	Grade 2	serous/papillary
VOC-A-193	**72.1**	**61.4**	IIA	Grade 3	serous/papillary
VOC-A-194	**94.5**	**5.0**	IIIC	Grade 3	serous/papillary
VOC-A-195	**23.6**	**5.7**	IIIC	Grade 3	serous/papillary
VOC-A-196	**19.9**	**8.1**	IIIC	Grade 2	serous/papillary
VOC-A-197	**58.6**	**6.1**	IIIA	Grade 3	serous/papillary
VOC-A-198	**90.1**	**4.9**	IIC	Grade 3	mixed
VOC-A-199	**75.2**	**3.0**	IIIC	Grade 1	serous/papillary
VOC-A-200	**23.6**	**15.1**	IV	Grade 2	serous/papillary
VOC-A-201	**NS**	**NS**	IIIC	Grade 2	serous/papillary
VOC-A-202	**86.0**	**18.9**	IB	Grade 1	serous/papillary
VOC-A-203	**45.5**	**11.3**	IC	Grade 1	serous/papillary
VOC-A-204	**NS**	**NS**	unstaged	Grade 1	serous/papillary
VOC-A-205	**90.2**	**41.3**	IIIC	Grade 3	serous/papillary
VOC-A-206	**71.4**	**34.9**	IA	Grade 1	serous/papillary
VOC-A-207	**NS**	**NS**	IIB	Grade 3	serous/papillary
VOC-A-208	**96.1**	**22.8**	IIIC	Grade 3	endometrioid
VOC-A-209	**81.3**	**66.3**	IC	Grade 1	endometrioid
VOC-A-210	**4.9**	**95.5**	IC	Grade 3	clear cell
VOC-A-212	**0.9**	**8.7**	IIIC	Grade 3	other
VOC-A-214	**0.1**	**44.8**	IIIC	Grade 3	N/A
VOC-A-215	**0.0**	**52.6**	IIIC	Grade 3	mucinous
VOC-A-216	**0.2**	**66.6**	IC	Grade 1	mucinous
VOC-A-217	**65.7**	**41.9**	IA	Grade 1	serous/papillary
VOC-A-218	**98.5**	**54.7**	IC	Grade 1	serous/papillary
VOC-A-219	**71.3**	**18.4**	IV	Grade 3	serous/papillary
VOC-A-221	**48.7**	**18.0**	IIIC	Grade 3	serous/papillary
VOC-A-222	**77.9**	**2.6**	IIIC	Grade 3	serous/papillary
VOC-A-223	**36.5**	**57.6**	IV	Grade 3	serous/papillary
VOC-A-224	**28.1**	**17.0**	IIIC	Grade 2	serous/papillary
VOC-A-225	**18.3**	**79.0**	IC	Grade 2	serous/papillary
VOC-A-226	**40.8**	**4.9**	IIIC	Grade 3	serous/papillary
VOC-A-227	**NS**	**NS**	unstaged	Grade 2	serous/papillary

(*Continued*)

VOC-A-228	**35.5**	**12.0**	IIIC	Grade 2	serous/papillary
VOC-A-229	**NS**	**NS**	IIIC	Grade 1	serous/papillary
VOC-A-230	**13.3**	**30.2**	IV	Grade 3	serous/papillary
VOC-A-231	**0.4**	**31.7**	IC	Grade 2	endometrioid
VOC-A-232	**31.5**	**6.4**	IIIC	Grade 3	endometrioid
VOC-A-233	**3.8**	**65.2**	IIIC	Grade 2	endometrioid
VOC-A-234	**22.9**	**3.7**	IIIA	Grade 3	endometrioid
VOC-A-236	**1.5**	**7.7**	IIIC	Grade 3	clear cell
VOC-A-237	**80.8**	**6.8**	IIIC	Grade 2	serous/papillary
VOC-A-238	**20.7**	**93.1**	IA	Grade 1	serous/papillary
VOC-A-239	**83.0**	**93.4**	IC	Grade 1	serous/papillary
VOC-A-240	**2.7**	**71.1**	IIA	Grade 3	endometrioid
VOC-A-241	**56.1**	**31.4**	IIIC	Grade 1	serous/papillary
VOC-A-242	**0.0**	**1.9**	IIIA	Grade 3	endometrioid
VOC-A-243	**23.9**	**3.0**	IIIC	Grade 2	serous/papillary
VOC-A-244	**38.1**	**7.8**	IIIC	Grade 3	serous/papillary
VOC-A-245	**73.0**	**10.5**	IV	Grade 3	serous/papillary
VOC-A-246	**100**	**25.6**	IIIC	Grade 2	serous/papillary
VOC-A-247	**22.9**	**4.1**	IIIC	Grade 2	serous/papillary
VOC-A-248	**5.0**	**3.1**	IIIC	Grade 3	endometrioid
VOC-A-249	**NS**	**NS**	IIIC	Grade 3	serous/papillary
VOC-A-250	**21.8**	**8.0**	IIIB	Grade 3	serous/papillary
VOC-A-251	**41.9**	**5.8**	IIIC	Grade 2	serous/papillary
VOC-A-252	**77.9**	**18.4**	IIIC	Grade 2	serous/papillary
VOC-A-253	**38.4**	**10.5**	IIIC	Grade 3	serous/papillary
VOC-A-254	**23.1**	**2.9**	IIIC	Grade 2	serous/papillary
VOC-A-255	**64.5**	**6.7**	IIIC	Grade 3	serous/papillary
VOC-A-256	**9.1**	**4.0**	IIIC	Grade 3	other
VOC-A-257	**44.2**	**16.4**	IV	Grade 3	serous/papillary
VOC-A-258	**4.6**	**58.9**	IA	Grade 2	mucinous
VOC-A-259	**80.5**	**67.0**	IIIA	Grade 1	serous/papillary
VOC-A-260	**17.8**	**8.7**	IIIC	Grade 1	serous/papillary
VOC-A-261	**10.7**	**66.9**	IIIA	Grade 1	mixed
VOC-A-262	**68.6**	**37.1**	IV	Grade 3	serous/papillary
VOC-A-263	**47.6**	**70.9**	IA	Grade 1	serous/papillary
VOC-A-264	**21.1**	**26.6**	IC	Grade 2	serous/papillary
VOC-A-265	**15.7**	**22.7**	IC	Grade 2	endometrioid
VOC-A-266	**66.8**	**47.0**	IA	Grade 1	serous/papillary

(*Continued*)

VOC-A-267	**73.8**	**1.9**	IIIC	Grade 3	serous/papillary
VOC-A-268	**14.9**	**66.5**	IIIC	Grade 3	serous/papillary
VOC-A-269	**0.3**	**10.1**	IA	Grade 2	endometrioid
VOC-A-270	**99.7**	**83.7**	IIIC	Grade 3	serous/papillary
VOC-A-271	**26.6**	**30.8**	IIIC	Grade 2	serous/papillary
VOC-A-272	**0.9**	**2.1**	IA	Grade 1	mucinous
VOC-A-273	**92**	**78**	IIIC	Grade 3	serous/papillary
VOC-A-274	**1.8**	**4.7**	IA	Grade 3	other
VOC-A-275	**89.0**	**71.6**	IC	Grade 1	serous/papillary
VOC-A-276	**98.2**	**5.9**	IIIC	Grade 2	serous/papillary
VOC-A-277	**4.1**	**20.8**	IIIC	Grade 3	endometrioid
VOC-A-278	**26.0**	**2.6**	IIIC	Grade 2	serous/papillary
VOC-A-279	**58.5**	**31.0**	IIIC	Grade 3	serous/papillary
VOC-A-280	**69.0**	**33.7**	IIIC	Grade 2	serous/papillary
VOC-A-281	**53.4**	**6.0**	IV	Grade 3	serous/papillary
VOC-A-282	**100**	**23.9**	IIIC	Grade 2	serous/papillary
VOC-A-283	**98.5**	**80.9**	IIIC	Grade 3	mixed
VOC-A-284	**14.0**	**2.8**	IIIC	Grade 3	serous/papillary
VOC-A-285	**4.8**	**5.3**	IIIC	Grade 3	N/A
VOC-A-286	**0.4**	**89.0**	IIIC	Grade 2	mucinous
VOC-A-287	**12.1**	**5.9**	IIIC	Grade 3	serous/papillary

Values are percentage of tumor cells staining positive for COX-1 or COX-2 by automated image analysis. NS = no spot. N/A represent tumors of non-epithelial origin.

Supplementary Table S4: List of Taqman used for QPCR analysis

Gene symbol	Common gene name	Life Technologies identifier
PTGS1	COX-1	Hs00377726_m1
PTGS2	COX-2	Hs01573469_m1
CDKN1A	p21	Hs00355782_m1
CCND1	cyclin D1	Hs00765553_m1
VEGFC	VEGFC	Hs00153458_m1
CDH2	N-cadherin	Hs00983056_m1
SNAI1	SNAIL	Hs00195591_m1
MMP7	MMP7	Hs01042796_m1
MMP2	MMP2	Hs01548727_m1
FN1	Fibronectin	Hs00365052_m1
CDH1	E-cadherin	Hs01023894_m1
GAPDH	GAPDH	Hs02758991_g1
